# Integrated model based on ultrasound attenuation and metabolic biomarkers for noninvasive assessment of hepatic fat fraction categories in MASLD: a QCT-referenced study

**DOI:** 10.3389/fphys.2026.1804061

**Published:** 2026-05-29

**Authors:** Zejun Ma, Hongbin Wang, Xiaojie Sun, Shuyu Zhou, Ye Sun, Xiaoguang Wang, Tao Li, Xiaohe Yang, Jiancheng Xu, Wanjun Guo, Meng Wang, Alfred Wei Chieh Kow, Huimao Zhang, Lei Zhang, Xiaofeng Sun

**Affiliations:** 1 Department of Cadre’s Wards Ultrasound Diagnostics, Ultrasound Diagnostic Center, The First Hospital of Jilin University, Changchun, China; 2 Department of Radiology, First Hospital of Jilin University, Changchun, China; 3 China-Singapore Belt and Road Joint Laboratory on Liver Disease research, Changchun, China; 4 The First Hospital of Jilin University, Changchun, China; 5 Division of Hepatobiliary & Pancreatic Surgery, Department of Surgery, National University Hospital Singapore, Singapore, Singapore; 6 Department of Surgery, Yong Loo Lin School of Medicine, National University of Singapore, Singapore, Singapore; 7 Division of Surgical Oncology, National University Cancer Institute Singapore (NCIS), National University Health System, Singapore, Singapore; 8 National University Digestive Center (NUCD), National University Health System Singapore, Singapore, Singapore

**Keywords:** biochemical markers, hepatic steatosis, MASLD, QCT (quantitative computed tomography), serum ferritin, USAT

## Abstract

**Background:**

Hepatic steatosis is a common metabolic disorder for which accessible noninvasive assessment remains clinically relevant. This study aimed to evaluate the QCT-referenced performance of ultrasound attenuation (USAT) and an integrated model combining metabolic biomarkers for non-invasive categorization according to hepatic fat fraction in metabolic dysfunction-associated steatotic liver disease (MASLD), using quantitative computed tomography (QCT)-derived hepatic fat fraction categories as pragmatic imaging comparator labels.

**Methods:**

A total of 172 participants were enrolled and categorized into hepatic fat fraction categories by QCT. USAT values, along with serum levels of ALT, AST, and ferritin, were collected. Three models were evaluated: USAT-only, laboratory-only, and an integrated USAT + laboratory model. Model performance was assessed using fold-separated internal validation, including nested five-fold stratified cross-validation with feature selection performed within training folds. Harrell’s optimism-corrected bootstrap analysis was also performed as a supplementary internal validation method.

**Results:**

USAT values increased significantly with hepatic fat fraction categories (P<0.001). In fold-separated internal validation, the USAT-only model achieved an AUC of 0.847 (95% CI, 0.780–0.902) for QCT-referenced detection of imaging-defined steatosis, while the laboratory-only model achieved an AUC of 0.753 (95% CI, 0.665–0.829). A fixed integrated model including USAT, ALT, and ferritin achieved an AUC of 0.845 (95% CI, 0.772–0.906), but did not significantly improve AUC compared with USAT alone. Multiclass categorization remained exploratory and limited, particularly for Category 2, which included only 31 participants and showed weak discrimination in the corrected random forest analysis (AUC=0.569 for the USAT + ALT + ferritin model). Subgroup analyses showed higher performance in females, younger participants, and those with higher BMI.

**Conclusion:**

USAT shows promise as a noninvasive adjunct for QCT-referenced detection of imaging-defined hepatic steatosis in a single-center Chinese health-examination cohort, pending external validation. Adding ALT and ferritin may improve sensitivity and calibration, but did not significantly improve AUC over USAT alone. Because discrimination for the intermediate Category 2 group remained weak, the current model should not be considered reliable for full hepatic fat fraction categorization. Further validation in external, multicenter, and more diverse populations is required before any clinical use.

## 1 Introduction

Metabolic dysfunction–associated steatotic liver disease (MASLD) has emerged as one of the most prevalent chronic liver diseases worldwide, affecting approximately 30% of the adult population and continuing to increase in prevalence in parallel with the global rise in metabolic disorders (Shi et al., 2021; Wang et al., 2022; Yang et al., 2024). MASLD is closely associated with various metabolic disorders, including type 2 diabetes mellitus (T2DM), cardiovascular disease, and chronic kidney disease, underscoring its systemic nature beyond hepatic involvement (Eslam et al., 2020a; Rinella et al., 2023). Rather than being confined to the liver, MASLD is now recognized as a hepatic manifestation of widespread metabolic dysregulation, posing significant long-term health risks. In China, MASLD represents a major and growing public health burden, with large population-based studies showing a high prevalence in the adult population (Shi et al., 2021; Wang et al., 2022; Yang et al., 2024).

With the rising global burden of metabolic syndrome, the etiology of steatotic liver disease has shifted from traditional alcohol-related causes to a complex interplay of metabolic abnormalities. In 2020, an international expert consensus proposed the term metabolic dysfunction-associated fatty liver disease (MAFLD) to emphasize the metabolic basis of the disease (Eslam et al., 2020a). More recently, the 2023 multisociety nomenclature update introduced metabolic dysfunction-associated steatotic liver disease (MASLD) (Rinella et al., 2023; EASL-EASD-EASO, 2024; Tilg et al., 2026). In the present study, we use MASLD throughout the manuscript in accordance with the current consensus terminology.

The pathogenesis of MASLD is multifactorial, involving not only hepatic steatosis but also progression to hepatocellular injury, inflammatory activity, and fibrotic remodeling (Tilg and Effenberger, 2020). These changes may eventually lead to metabolic dysfunction–associated steatohepatitis (MASH), cirrhosis, or hepatocellular carcinoma. Clinically, early MASLD is often asymptomatic or presents with nonspecific features, such as mildly elevated aminotransferase levels or increased echogenicity on ultrasound, which are frequently overlooked. Given its progressive yet potentially reversible nature, timely identification and intervention in the early stages of MASLD are essential to reduce the risk of advanced fibrosis and systemic metabolic complications (Eslam et al., 2020b). This highlights the urgent need for a noninvasive, accessible, cost-effective, and sensitive diagnostic strategy to support primary prevention and longitudinal disease management.

Liver biopsy has long served as the reference standard for diagnosing and staging steatotic liver disease, including MASLD (Spearman et al., 2021). However, its invasive nature, sampling variability, high cost, and limited patient acceptance make it impractical for routine screening or longitudinal follow-up in clinical practice. In recent years, magnetic resonance imaging-derived proton density fat fraction (MRI-PDFF) has been widely recognized as a noninvasive reference standard for the quantitative assessment of hepatic steatosis, owing to its high accuracy and reproducibility. It has been widely used as a benchmark in validating novel imaging modalities (Stine et al., 2021; Tamaki et al., 2022). Despite its excellent diagnostic performance, the high cost, complexity, and limited availability of MRI-PDFF restrict its use in primary care settings and large-scale population screening (Tamaki et al., 2022).

As a more accessible alternative, quantitative computed tomography (QCT) estimates hepatic fat burden by assessing parenchymal density. Quantitative computed tomography (QCT) has demonstrated strong correlation with proton density fat fraction (PDFF) measurements, as supported by several large-scale validation studies and systematic reviews (Xu et al., 2018; Guo et al., 2020). These studies have confirmed that QCT provides a reliable and cost-effective alternative to PDFF for hepatic fat quantification, with comparable diagnostic performance and lower radiation exposure. Importantly, QCT is increasingly used in health check-up settings, where it provides a feasible and scalable reference for validating new imaging tools (Starekova et al., 2021).

Conventional ultrasound is widely available and frequently used in routine practice to detect fatty liver, but its accuracy is highly operator-dependent and generally insufficient for reliable categorization (Wang et al., 2024). In this context, the ultrasound attenuation parameter (USAT) has emerged as a promising quantitative technique (Ferraioli et al., 2024; Naghibi et al., 2026). By measuring the degree of signal attenuation within liver tissue, USAT enables rapid, cost-effective, and reproducible fat quantification. Its ease of use and real-time feedback make it particularly appealing for screening high-risk populations and for use in resource-limited settings (Bende et al., 2021). Nonetheless, further validation of USAT against established reference standards such as QCT or MRI-PDFF remains warranted, particularly in broader clinical settings and diverse study populations (Qi et al., 2025). Moreover, it remains unclear whether diagnostic performance can be further improved by integrating USAT with metabolic and biochemical markers (Jiang et al., 2023; Cannella et al., 2025).

On the basis of this background, this study aimed to evaluate the association and QCT-referenced classification performance of USAT for detecting imaging-defined hepatic steatosis in a health examination setting. Although MRI-PDFF is widely recognized as a noninvasive reference standard for hepatic fat quantification, its high cost and limited accessibility restrict its use in large-scale screening and routine clinical practice. In contrast, QCT is more readily available in health examination settings and provides a quantitative and standardized assessment of hepatic fat content. Therefore, in the present study, QCT was selected as a pragmatic imaging comparator for model development and internal validation, rather than as a standalone gold standard equivalent to histology or MRI-PDFF. In addition to USAT, anthropometric features and metabolic biomarkers were explored in multivariable models to evaluate whether they provided adjunctive information beyond imaging alone. Furthermore, a composite risk score derived from this integrated model was explored in relation to other metabolic comorbidities, including dysglycemia, dyslipidemia, and insulin resistance, to explore the broader relevance of imaging-based MASLD assessment in the context of systemic metabolic dysfunction.

Overall, this study seeks to explore a practical and noninvasive QCT-referenced approach for detecting imaging-defined hepatic steatosis and for preliminary hepatic fat fraction categorization. Given the anticipated difficulty in distinguishing intermediate hepatic fat categories, the findings should be interpreted as exploratory and hypothesis-generating rather than as evidence of a clinically deployable categorization tool.

## 2 Materials and methods

### 2.1 Study design and participants

This prospective, single-center, cross-sectional study was conducted at the First Hospital of Jilin University from September 2023 to March 2025. Adult participants undergoing liver quantitative computed tomography (QCT) for clinical or screening purposes were consecutively enrolled. Venous blood samples were collected within the same assessment window as the imaging examinations. The study protocol was approved by the institutional ethics committee, and written informed consent was obtained from all participants prior to enrollment (Approval No. 24K183-001).

### 2.2 Inclusion and exclusion criteria

Eligible participants met the following criteria: (a) Chinese citizens aged 18 years or older; (b) willingness to participate and provide written informed consent; (c) The MASLD group must meet the diagnostic criteria for metabolic dysfunction-related fatty liver disease (MASLD); (d) ability to complete standard ultrasound, USAT imaging, and QCT with adequate image quality; and (e) availability of liver function and related laboratory tests within 7 days of imaging.

Exclusion criteria included: (a) recent history of trauma, surgery, or other major invasive procedures; (b) prior chemotherapy, radiotherapy, or history of hepatic malignancy; (c) presence of severe organ-specific diseases, such as cardiac disorders (e.g., heart failure or coronary artery disease), hepatic conditions (e.g., cirrhosis or hepatocellular carcinoma), or pulmonary disease; (d) known malignancies, autoimmune disorders, or endocrine diseases that may significantly affect systemic metabolism; (e) recent (within 6 months) use of antiviral agents, hepatoprotective drugs, or medications that may alter liver function; (f) pregnancy or lactation; and (g) inability to complete imaging or laboratory assessments, or poor compliance with study procedures.

Available clinical records were reviewed for conditions that may confound ferritin interpretation, including clinically evident acute inflammation, known hemochromatosis, and recent blood transfusion. CRP was measured as an available inflammatory marker and was considered in exploratory analyses.

All participants underwent standardized clinical evaluation, including medical history collection, physical examination, laboratory testing, USAT assessment, and QCT imaging. Available medical history was reviewed for alcohol intake and medication exposure. Particular attention was given to medications that may affect hepatic fat accumulation or liver biochemistry, including statins, metformin, tamoxifen, and corticosteroids. Menopausal status was also reviewed in female participants when available. However, these variables were not completely available for all participants and therefore were not included as formal covariates in the main multivariable models. In the present study, hepatic steatosis for diagnostic modeling was defined based on QCT findings, with QCT category ≥1 indicating the presence of steatosis. According to current consensus definitions, MASLD requires hepatic steatosis in the presence of cardiometabolic risk factors (Rinella et al., 2023; van Erpecum et al., 2023). Therefore, participants with QCT-defined steatosis were considered as having imaging-defined MASLD for the purpose of analysis.

The intended study population should therefore be understood as Chinese adults undergoing health examination or clinical screening in a tertiary-care setting who were able to complete both USAT and QCT examinations with adequate image quality and who did not meet the major exclusion criteria listed above. The findings should not be directly extrapolated to community-based populations, non-Chinese or ethnically diverse populations, patients with established cardiovascular disease, autoimmune disease, malignancy, cirrhosis, hepatocellular carcinoma, severe systemic illness, pregnancy or lactation, or individuals receiving medications that may substantially affect liver fat content or liver biochemistry. Generalizability to other ultrasound vendors, acquisition protocols, and primary-care settings requires external validation.

### 2.3 Imaging examinations

#### 2.3.1 Ultrasound attenuation imaging

All participants underwent ultrasound attenuation (USAT) imaging within 3 days of QCT examination. The examinations were scheduled within the same short assessment window whenever possible, and participants with acute illness, major invasive procedures, or incomplete imaging/laboratory assessment were excluded. The exact hour-level interval between USAT and QCT was not available for all participants and is therefore acknowledged as a limitation.

All USAT examinations were performed by a single senior ultrasound physician with more than 5 years of experience in abdominal ultrasound, using a standardized ultrasound system (Resona R9 Pro; Mindray, China) equipped with a convex array transducer capable of USAT measurements (frequency range: 1–6 MHz). Before study initiation, the operator followed a standardized acquisition protocol, including patient positioning, intercostal scanning approach, ROI placement, quality-control criteria, and measurement recording. Because all USAT examinations were performed by one operator, inter-operator reproducibility could not be assessed in the present study.

Before the examination, participants fasted for at least 6 hours. USAT measurements were acquired with the patient in the supine or left lateral decubitus position, using an intercostal acoustic window to access segment 5 (S5) of the right hepatic lobe. A region of interest was carefully selected to avoid large vessels and artifacts. For each participant, five valid measurements were obtained, and the median value was recorded as the final attenuation coefficient (expressed in dB/cm/MHz). Measurements failing to meet the system-defined signal quality threshold were excluded from analysis. The USAT operator were blinded to QCT results throughout the procedure. For each participant, five valid measurements were obtained and the median value was used as the final USAT value to reduce within-examination variability. However, a formal intra-operator reproducibility study based on repeated examinations in an independent subset was not performed.

#### 2.3.2 Quantitative computed tomography

QCT was performed using a multi-detector spiral CT system (Siemens SOMATOM Force CT; Siemens Healthineers, Germany) according to the institutional liver QCT protocol. Participants were positioned supine, and the scanning range encompassed the entire liver. The primary analysis focused on segment 5 of the right hepatic lobe to match the USAT acquisition region as closely as possible. QCT images were transferred to dedicated QCT software (Mindways Software Inc., USA) for quantitative hepatic fat fraction analysis. Because the retrospective DICOM-level extraction of tube voltage, tube current, reconstruction kernel, slice thickness, and phantom calibration details was not complete for all participants at the time of revision, these technical parameters could not be reliably summarized for the full cohort.

Standardized circular ROIs with a diameter of approximately 1.5cm were placed in homogeneous liver parenchyma. ROI placement avoided large vessels, bile ducts, focal lesions, visible artifacts, and the liver margin. Multiple valid hepatic ROIs were used, and the mean hepatic fat fraction was calculated as the participant-level QCT value. All imaging data were independently reviewed by two experienced radiologists. In cases of discrepancy, a third radiologist adjudicated the measurements, and a consensus reading was used for final analysis.

Quantitative analysis was conducted using dedicated software (Mindways Software Inc., USA). Standardized regions of interest (ROIs) with a diameter of 1.5cm were placed in multiple hepatic locations to measure liver fat content. The average hepatic fat fraction was calculated across all ROIs.

A mean hepatic fat fraction ≥5% was considered indicative of steatosis. This threshold was adopted with reference to histological definitions of hepatic steatosis and is consistent with commonly used MRI-PDFF–based diagnostic frameworks, in which a fat fraction of approximately 5% is widely regarded as the lower limit for steatosis detection.

To our knowledge, there is currently no universally accepted or fully validated QCT-specific categorization system for hepatic hepatic fat fraction categories. Therefore, in the present study, hepatic fat fraction category was performed using a pragmatic, study-specific operational approach based on hepatic fat fraction.

A mean hepatic fat fraction ≥5% was considered indicative of steatosis. This threshold was adopted with reference to established histological definitions of hepatic steatosis and MRI-PDFF–based diagnostic frameworks, in which approximately 5% fat fraction is commonly used as the lower threshold for steatosis detection. To our knowledge, however, there is no universally accepted or fully validated QCT-specific grading system for hepatic steatosis severity. Therefore, the additional cutoffs of 10% and 25% were used as study-specific operational thresholds to stratify mild, intermediate, and higher hepatic fat fraction ranges for exploratory analysis. These categories should be interpreted as QCT-derived operational strata for research purposes rather than as validated clinical severity grades.

The predefined categorization scheme was applied consistently across all analyses and should be interpreted as an operational stratification for research purposes. The categories are defined operationally based on QCT-derived hepatic fat fraction and are for research purposes; they do not constitute a validated clinical grading system.

The mean hepatic fat fraction for each participant was recorded and used for both categorical categorization and continuous analysis. Descriptive statistics including mean ± SD and interquartile ranges (IQRs) for hepatic fat fraction were calculated for each QCT category.

All imaging data were independently analyzed by two experienced radiologists. In cases of discrepancy, a third radiologist reviewed the measurements, and a consensus reading was used for final analysis. Although formal intraobserver and interobserver agreement analyses were not performed, standardized acquisition protocols and double-reading procedures with consensus resolution were applied to minimize measurement variability.

#### 2.3.3 Laboratory assessments

Venous blood samples were collected within the same assessment window as the imaging examinations. Standardized biochemical testing was performed to evaluate multiple clinical parameters, including:

Liver function markers: aspartate aminotransferase(AST), alanine aminotransferase (ALT), total bilirubin, γ-glutamyl transpeptidase (GGT), and alkaline phosphatase (ALP);Lipid profile: total cholesterol (TC), low-density lipoprotein (LDL), high-density lipoprotein (HDL), and triglycerides (TG);Glucose metabolism: fasting plasma glucose (FPG) and glycated hemoglobin (HbA1c);Renal function: serum creatinine (Scr) and uric acid.

Blood samples were processed immediately and analyzed in the hospital’s central laboratory. All assays were conducted by trained laboratory personnel using validated and standardized equipment.

ALT was measured in the hospital central laboratory using an automated biochemical analyzer according to standardized clinical laboratory procedures. Sex-specific laboratory reference ranges and analytical coefficients of variation should be reported according to the institutional quality-control records where available.

To explore the potential mechanistic contribution of metabolic markers in MASLD detection, biochemical indices were categorized into four functional groups: (1) liver function group (ALT, AST, GGT, ALP); (2) metabolic group (fasting insulin, C-peptide, HOMA-IR, TG); (3) renal function group (serum creatinine, uric acid); and (4) inflammation and iron metabolism group (C-reactive protein [CRP], ferritin).

### 2.4 Reporting guideline compliance

This manuscript was prepared with reference to the TRIPOD+AI reporting guideline for clinical prediction models using regression and machine-learning methods. Because the study evaluated QCT-referenced detection and categorization of imaging-defined hepatic steatosis and retained diagnostic-accuracy terminology in parts of the analysis, relevant items from the STARD 2015 guideline were also considered. Completed TRIPOD+AI and STARD 2015 checklists are provided as **Supplementary Files**. The present study should be interpreted as a QCT-referenced prediction-model and method-comparison study rather than as a definitive diagnostic accuracy study against a histological or MRI-PDFF gold standard.

### 2.5 Statistical analysis

All statistical analyses were performed using Python (version 3.10), with key packages including pandas, scikit-learn, numpy, and matplotlib. Continuous variables were summarized as mean ± standard deviation or as median with interquartile range (IQR), depending on distributional characteristics. Categorical variables were reported as counts and percentages.

No missing values were present in the final analytic dataset for the variables used in the main models.

To assess QCT-referenced classification performance for imaging-defined steatosis, binary logistic regression models were constructed using QCT category ≥1 as the comparator definition. Three models were evaluated for QCT-referenced detection of imaging-defined steatosis: (i) a USAT-only model, (ii) a laboratory-only baseline model incorporating routinely available biochemical indicators, and (iii) an integrated USAT + laboratory model. To address selection-induced optimism, model validation was re-performed using nested five-fold stratified cross-validation. In each outer fold, preprocessing, variable screening, feature selection, and model fitting were conducted exclusively within the training subset, and the held-out fold was used only for performance assessment.

Model performance was assessed using discrimination and calibration metrics, including AUC, sensitivity, specificity, accuracy, F1 score, and Brier score. Calibration was further evaluated using calibration plots based on out-of-fold predicted probabilities. For binary models, observed versus predicted probabilities were plotted across risk strata. For multiclass analyses, class-specific calibration was considered exploratory because of the limited sample sizes in Category 2 and Category 3. Bootstrap resampling with 2,000 replicates was used to derive 95% confidence intervals for AUC. In addition, Harrell’s optimism-corrected bootstrap analysis was performed as a supplementary internal validation method. Optimal probability thresholds were identified using the Youden index. Sensitivity and specificity were calculated at the corresponding cut-off points. Pairwise AUC differences between models were evaluated using the DeLong test and paired bootstrap resampling based on out-of-fold predictions. Because no *a priori* sample-size calculation was performed, a *post hoc* precision analysis was conducted by reporting the width and half-width of 95% confidence intervals for key AUC estimates. Class distribution was explicitly examined to assess the stability of multiclass classification, particularly for Category 2 and Category 3. The events-per-variable ratio was evaluated for the initial candidate predictor set and for the reduced prespecified models. Model complexity and approximate events-per-variable considerations are summarized in **Supplementary Table 1**. Because the initial laboratory-only model included 17 candidate biochemical predictors, this model was treated as an exploratory benchmark rather than as a final parsimonious prediction model. Sensitivity analyses were performed using simpler prespecified models, including USAT-only and a fixed integrated model including USAT, ALT, and ferritin.

Exploratory decision-curve analysis was performed for the binary QCT-referenced detection models to estimate net benefit across a range of threshold probabilities. Because the study lacked external validation and the outcome was QCT-referenced rather than based on histology or MRI-PDFF, decision-curve analysis was interpreted cautiously. Net reclassification improvement was not calculated because no prespecified clinically validated risk categories were available for the QCT-referenced outcome, and the integrated model did not significantly improve AUC over USAT alone.

Ordinal logistic regression was employed to analyze associations between biochemical markers and MASLD hepatic fat fraction category. For interpretability, a multinomial logistic regression model was additionally fitted using QCT category 0 as the reference category. Regression coefficients, odds ratios, 95% confidence intervals, and P values were reported for Category 1, Category 2, and Category 3 versus Category 0. This analysis was intended to provide category-specific interpretation rather than to replace the ordinal regression or fold-separated prediction analyses. To evaluate potential confounding by adiposity, an additional adjusted ordinal logistic regression model was constructed including USAT, ALT, ferritin, BMI, and waist-to-hip ratio. BMI was used as an indicator of general adiposity, and waist-to-hip ratio was used as an available surrogate of central adiposity because waist circumference was not available as a complete standalone variable in the present dataset. Variance inflation factors (VIFs) were calculated to assess multicollinearity among predictors in the adjusted integrated model. In addition, exploratory three-class random forest models were developed to classify participants with QCT-defined steatosis into Category 1, Category 2, and Category 3. Category 0 was not included in this multiclass random forest analysis because binary steatosis detection was evaluated separately using logistic regression models. Random forest performance was evaluated using strict five-fold stratified cross-validation. In each fold, model fitting was performed exclusively within the training subset, and the held-out validation fold was used only once for performance assessment. No validation-fold information was used during model fitting. Accuracy, macro-averaged F1 score, macro-AUC, and class-specific one-vs-rest AUCs were calculated from pooled out-of-fold predictions rather than from training-set predictions.

For between-category comparisons of baseline and biochemical variables, effect sizes were reported in addition to P values. For continuous variables, the maximum absolute pairwise standardized mean difference across QCT-derived categories was calculated. For categorical variables, Cramér’s V was used as the effect-size measure. To account for multiple univariable comparisons, Benjamini–Hochberg false discovery rate correction was applied. Detailed effect sizes and FDR-adjusted P values are provided in **Supplementary Table 3**.

Feature importance scores derived from the random forest model were used to interpret the relative contribution of predictors within the integrated diagnostic framework. Data distribution was assessed using the Shapiro–Wilk test.

Reproducibility analysis (e.g., intraclass correlation coefficients) was not conducted in the present study and will be addressed in future work.

For between-category comparisons of baseline and biochemical variables, effect sizes were reported in addition to P values. Standardized mean differences or rank-based effect-size measures were calculated as appropriate according to variable distribution. To account for multiple univariable comparisons, Benjamini–Hochberg false discovery rate correction was applied, and FDR-adjusted P values were reported.

All statistical tests were two-sided, and a p-value < 0.05 was considered statistically significant.

Net reclassification improvement was not calculated because no prespecified clinically validated risk categories were available for the QCT-referenced outcome, and the integrated model did not significantly improve AUC over USAT alone.

## 3 Results

### 3.1 Participant characteristics

**Figure 1:**
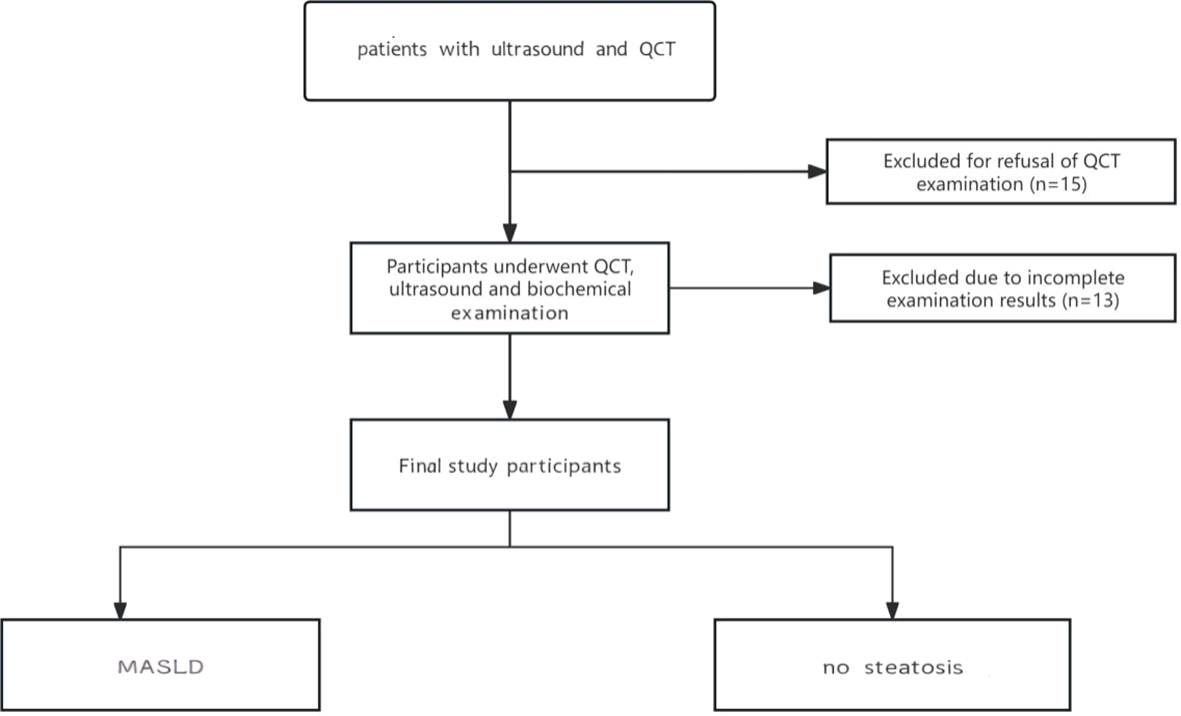
STROBE/TRIPOD-style participant flow diagram. A total of 200 participants were screened. Fifteen were excluded because of refusal to undergo QCT examination, and 13 were excluded because of incomplete laboratory or imaging data. The final complete-case analytic cohort included 172 participants.

A total of 200 adult participants were screened. Among them, 15 were excluded because they refused QCT examination, and 13 were excluded because of incomplete laboratory or imaging data. A STROBE/TRIPOD-style participant flow diagram is shown in **Figure 1**.

Ultimately, 172 participants (median age, 44 years; IQR, 34–59 years) were included in the final analysis. Based on QCT-derived liver attenuation values, 52 participants were classified as category 0 (no steatosis), while 120 participants were classified as having steatosis (QCT category ≥1), including all defined hepatic fat fraction categories. For the purpose of diagnostic analysis, QCT category ≥1 was considered indicative of hepatic steatosis (i.e., imaging-defined MASLD), and these QCT-derived categories were used as operational comparator labels to evaluate the QCT-referenced performance of USAT for detecting and categorization steatosis (**Figure 1**).

**Figure 2:**
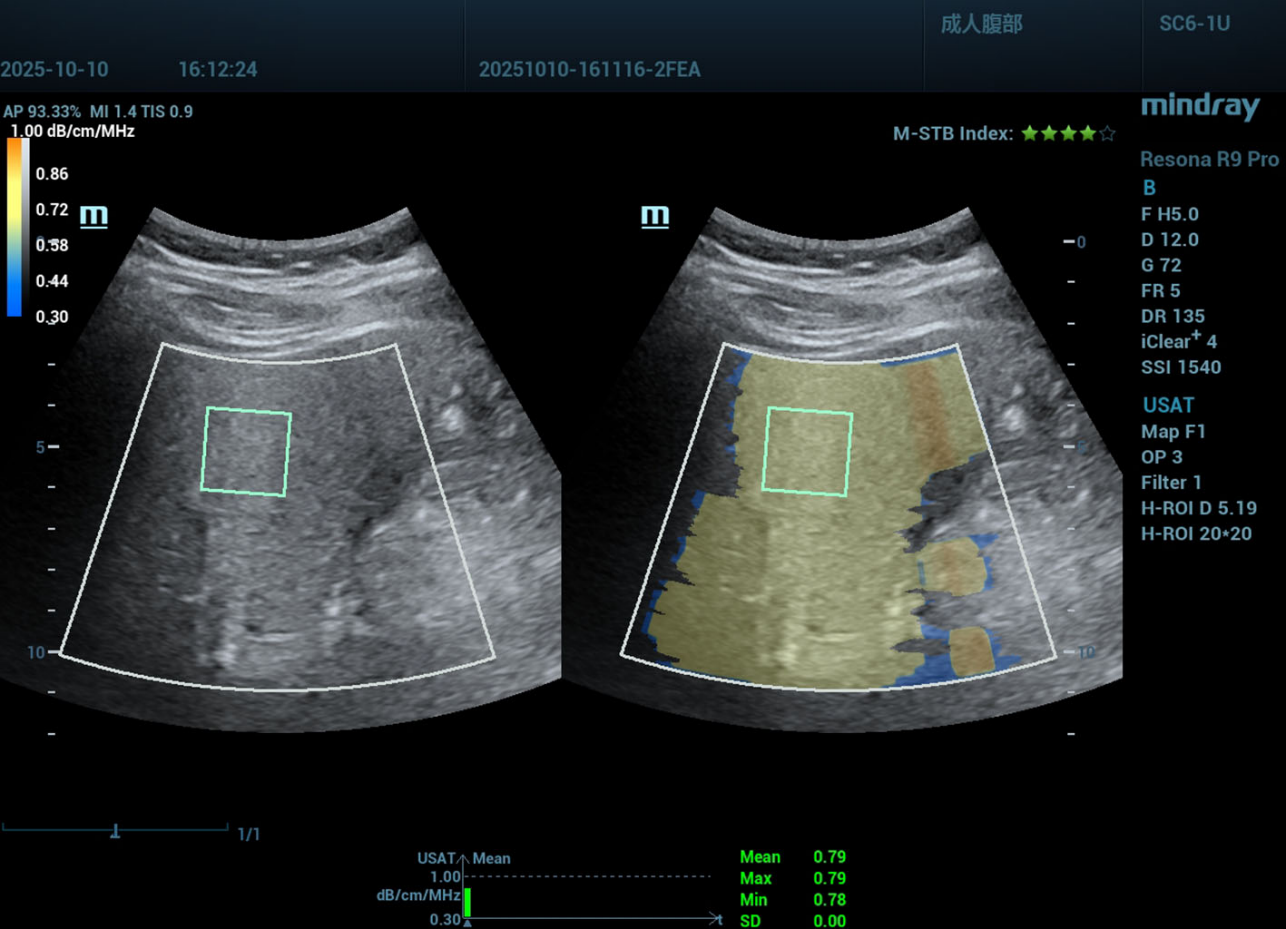
Representative images of multiparametric ultrasound attenuation (USAT) examination. USAT measurements were performed using a convex probe placed at segment 5 of the right hepatic lobe, avoiding major vessels and artifacts. The attenuation coefficient was automatically calculated based on the region of interest (ROI) selected within homogeneous liver parenchyma. The displayed image illustrates real-time attenuation mapping and signal quality indicators used for data validation.

Baseline demographic characteristics, biochemical profiles, and ultrasound attenuation results are summarized in **Table 1**. Representative USAT images are shown in **Figure 2**.

**Table 1:** summarizes the baseline demographic, biochemical, and ultrasound attenuation characteristics of the study population.

Characteristic	Median (IQR)
Age (years)	42.00 (35.75–52.25)
Height (cm)	168.00 (160.00–174.00)
Weight (kg)	78.00 (67.00–87.00)
BMI (kg/m²)	27.00 (24.11–29.63)
AST (U/L)	23.00 (19.70–29.85)
ALT (U/L)	27.05 (18.18–40.83)
GGT (U/L)	31.60 (21.48–49.17)
ALP (U/L)	72.10 (59.95–91.12)
Ferritin (ng/mL)	141.75 (77.12–230.50)
Iron (μmol/L)	21.55 (16.98–25.92)
TIBC (μmol/L)	62.00 (57.00–67.00)
CRP (mg/L)	0.81 (0.58–1.62)
Fasting Insulin (PU/L)	76.40 (54.59–100.17)
Fasting C-peptide (ng/mL)	0.83 (0.66–1.11)
USAT (dB/cm/MHz)	0.71 (0.59–0.81)
Systolic BP (mmHg)	128.00 (120.00–140.00)
Diastolic BP (mmHg)	80.00 (74.00–90.00)

Continuous variables are presented as median (interquartile range), and categorical variables are presented as number (%). MASH, metabolic dysfunction–associated steatohepatitis; MASLD, metabolic dysfunction–associated steatotic liver disease; USAT, ultrasound attenuation; QCT, quantitative computed tomography; ALT, alanine aminotransferase; AST, aspartate aminotransferase; GGT, γ-glutamyl transpeptidase; ALP, alkaline phosphatase; TG; triglycerides; LDL, low-density lipoprotein; HDL, high-density lipoprotein; FPG, fasting plasma glucose; HbA1c, glycated hemoglobin; CRP, C-reactive protein.

### 3.2 Comparison of clinical and biochemical parameters across QCT-based steatosis categories

Participant characteristics stratified by QCT-derived steatosis categories are summarized in **Table 2**. Multiple metabolic and liver function–related parameters differed significantly across the hepatic fat fraction category. The mean hepatic fat fraction measured by QCT increased progressively across categories, confirming the ordinal classification. category 0 participants had a mean of 3.2% (IQR 2.5–4.0%), category 1 had 6.8% (IQR 5.5–8.2%), category 2 had 15.3% (IQR 11.2–18.8%), and category 3 had 32.7% (IQR 26.0–38.5%). This presentation allows for a clearer understanding of the quantitative distribution underlying the categorical stratification.

**Table 2:** Summarizes clinical and biochemical parameters across QCT-defined steatosis categories in the study cohort.

Variable	No steatosis(IQR)	Category 1 median (IQR)	Category 2 median (IQR)	Category 3 median (IQR)	P
Age (years)	41.00 (32.75–49.25)	44.00 (36.00–54.00)	41.00 (38.50–53.00)	44.00 (36.00–52.00)	0.3235
BMI (kg/m²)	24.00 (21.89–26.25)	27.59 (25.99–29.87)	27.68 (24.82–32.95)	28.50 (26.50–31.05)	<0.0001
Weight (kg)	66.50 (59.75–75.15)	81.00 (72.88–88.22)	82.00 (72.50–91.50)	83.00 (75.00–88.00)	<0.0001
Waist-to-Hip Ratio	0.84 (0.79–0.90)	0.92 (0.85–0.95)	0.92 (0.84–0.96)	0.94 (0.88–0.99)	<0.0001
Height (m)	162.00 (155.75–172.25)	170.00 (164.00–174.00)	168.00 (162.50–174.50)	167.50 (164.00–174.00)	0.0184
Systolic BP (mmHg)	120.00 (119.50–127.00)	130.00 (124.50–140.00)	130.00 (123.50–140.00)	135.00 (125.00–140.00)	<0.0001
ALP (U/L)	61.30 (51.85–77.83)	77.15 (61.43–94.45)	70.50 (62.20–88.70)	81.00 (73.60–92.60)	0.0018
FastingC-peptide (ng/mL)	0.74 (0.62–0.89)	0.86 (0.66–1.11)	0.92 (0.71–1.26)	0.96 (0.69–1.23)	0.0036
Diastolic BP (mmHg)	77.50 (70.00–80.25)	80.00 (74.75–90.00)	80.00 (77.00–94.50)	80.00 (78.00–92.00)	0.0078
Fasting Insulin (PU/L)	68.62 (46.94–84.56)	73.53 (56.63–101.80)	96.35 (59.25–109.69)	92.30 (66.13–107.40)	0.0155
Uric Acid (UA) (μmol/L)	334.50 (271.50–406.50)	368.00 (331.50–441.50)	391.00 (339.00–456.00)	382.00 (291.00–472.00)	0.0156
Height (m)	162.00 (155.75–172.25)	170.00 (164.00–174.00)	168.00 (162.50–174.50)	167.50 (164.00–174.00)	0.0184
HDL (mmol/L)	1.31 (1.18–1.58)	1.23 (1.10–1.46)	1.26 (1.00–1.37)	1.18 (1.12–1.40)	0.0905
Fasting Plasma Glucose (FPG) (mmol/L)	5.28 (4.89–5.70)	5.38 (4.93–5.96)	5.60 (5.08–6.62)	5.77 (5.30–6.12)	0.1079
CRP (mg/L)	0.77 (0.42–1.29)	0.80 (0.59–1.53)	0.84 (0.67–1.90)	0.90 (0.60–2.22)	0.2408
Scr(μmol/L)	63.35 (54.70–71.80)	68.10 (59.85–75.80)	65.40 (57.30–74.40)	61.50 (59.40–71.60)	0.3080
LDL (mmol/L)	3.18 (2.88–3.55)	3.04 (2.55–3.65)	3.31 (3.06–3.62)	3.38 (2.59–3.84)	0.3142
Triglycerides (mmol/L)	1.12 (0.92–1.48)	1.49 (1.01–2.10)	2.06 (1.42–2.65)	1.76 (1.25–2.65)	0.0001
TC(mmol/L)	4.96 (4.60–5.43)	5.08 (4.53–5.69)	5.28 (4.54–5.91)	5.34 (4.04–5.79)	0.6993
Iron (μmol/L)	20.55 (16.68–25.90)	21.55 (18.50–25.62)	23.70 (16.75–26.85)	21.20 (16.89–25.70)	0.7985
TIBC (μmol/L)	62.64 (57.00–66.25)	62.80 (58.20–67.00)	60.90 (56.30–66.50)	61.00 (57.66–67.00)	0.9413
GGT (U/L)	20.85 (15.45–29.15)	34.60 (26.10–53.12)	38.10 (29.70–58.85)	39.30 (30.30–51.90)	<0.0001
ALT (U/L)	19.65 (13.60–26.23)	26.90 (18.12–37.72)	32.10 (25.85–41.95)	56.00 (29.00–70.30)	<0.0001
AST (U/L)	20.45 (17.25–22.95)	23.00 (18.90–27.07)	28.40 (22.20–34.70)	34.00 (23.00–40.70)	<0.0001
Ferritin (ng/mL)	86.72 (38.60–148.30)	147.95 (90.05–232.03)	173.70 (92.85–229.51)	218.60 (120.30–239.70)	<0.0001
USAT (dB/cm/MHz)	0.56 (0.50–0.65)	0.69 (0.62–0.76)	0.79 (0.71–0.84)	0.85 (0.77–0.95)	<0.0001
QCT Hepatic Fat Fraction (%)	3.2 ± 1.1 (IQR 2.5–4.0)	6.8 ± 1.4 (IQR 5.5–8.2)	15.3 ± 4.2 (IQR 11.2–18.8)	32.7 ± 6.1 (IQR 26.0–38.5)	

Participants were stratified according to QCT-based steatosis categories. The quantitative thresholds used for QCT categorization are described in the Methods section. Effect sizes and Benjamini–Hochberg FDR-adjusted P values for between-category comparisons are provided in **Supplementary Table 3**. Data are expressed as mean ± standard deviation, median (interquartile range), or number (%), as appropriate. QCT, quantitative computed tomography; MASLD, metabolic dysfunction–associated steatotic liver disease; USAT, ultrasound attenuation; BMI, body mass index; ALT, alanine aminotransferase; GGT, γ-glutamyl transpeptidase; ALP, alkaline phosphatase, HDL-C, high-density lipoprotein cholesterol. P values were calculated using analysis of variance (ANOVA) or Kruskal–Wallis tests for continuous variables and χ² tests for categorical variables’ values were calculated using Kruskal–Wallis tests for continuous variables and χ² tests for categorical variables. Detailed effect sizes and Benjamini–Hochberg FDR-adjusted P values are provided in **Supplementary Table 3**.

USAT values demonstrated a stepwise increase with higher steatosis categories (P <.0001), and body mass index (BMI) also rose significantly in parallel with hepatic fat fraction categories (P <0.0001). Serum alanine aminotransferase (ALT) and γ-glutamyl transpeptidase (GGT) levels were markedly elevated in participants with greater hepatic fat burden (both P <.0001). Markers associated with insulin resistance, including fasting insulin and fasting C-peptide, also varied significantly between groups (P=.0155 and P=.0036, respectively).

In addition, triglycerides (P=.0001) and alkaline phosphatase (ALP) (P=.0018) showed significant intergroup differences. Although changes in systolic and diastolic blood pressure were relatively modest, both reached statistical significance (P=.0309 and P=.0078, respectively). Other parameters, such as high-density lipoprotein cholesterol (HDL-C) and ferritin, did not differ significantly among QCT-defined steatosis categories.

These findings suggest that higher hepatic fat content, as assessed by QCT, is accompanied by progressive elevations in liver enzymes and worsening features of metabolic dysfunction.

### 3.3 Correlation between USAT and metabolic parameters

**Figure 3:**
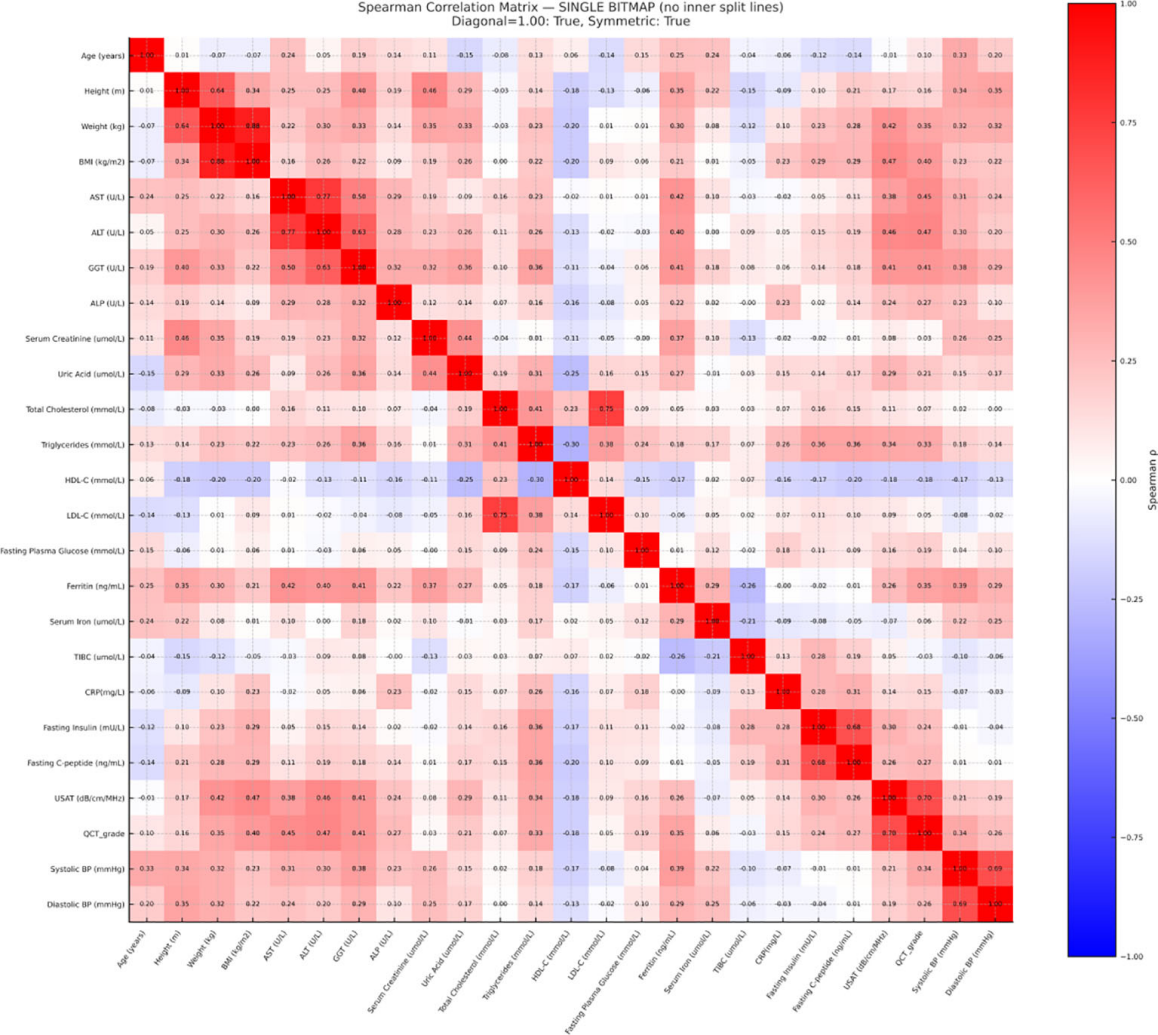
Spearman correlation matrix between USAT and metabolic, hepatic, and anthropometric parameters. The heatmap depicts the pairwise Spearman’s rank correlation coefficients (ρ) among the ultrasound attenuation (USAT) value and multiple clinical, biochemical, and anthropometric parameters in the study cohort. Color intensity reflects the strength and direction of the correlation (red: positive; blue: negative), with exact ρ values displayed in each cell. Variables include liver enzymes (ALT, AST, GGT, ALP), lipid profile (Triglycerides, HDL-C, LDL-C, Total Cholesterol), glycemic indices (Fasting Plasma Glucose, Fasting Insulin, Fasting C-peptide), iron metabolism markers (Ferritin, Serum Iron, TIBC), renal function parameters (Serum Creatinine, Uric Acid), inflammatory marker (CRP), and anthropometric measures (BMI, weight, height, waist-to-hip ratio, blood pressure).

To explore the relationship between ultrasound attenuation (USAT) and key clinical as well as metabolic parameters, Spearman’s rank correlation analysis was performed. As summarized in **Table 3** and **Figure 3**, USAT exhibited positive correlations with body mass index (BMI; ρ = 0.469, P < 0.0001), alanine aminotransferase (ALT; ρ = 0.464, P < 0.0001), body weight (ρ = 0.420, P < 0.0001), and waist-to-hip ratio (WHR; ρ = 0.397, P < 0.0001). These findings indicate that USAT was associated not only with QCT-derived hepatic fat fraction categories but also with adiposity-related anthropometric variables. Therefore, additional multivariable models adjusted for BMI and WHR were performed to evaluate whether USAT retained an independent association with QCT-derived hepatic fat fraction category after accounting for general and central adiposity.

**Table 3:** Correlation between USAT and clinical/metabolic parameters.

Variable	Spearman ρ	P value
BMI (kg/m²)	0.469	<0.0001
ALT (U/L)	0.464	<0.0001
Weight (kg)	0.420	<0.0001
GGT (U/L)	0.413	<0.0001
Waist-to-Hip Ratio	0.397	<0.0001
AST (U/L)	0.375	<0.0001
TG (mmol/L)	0.34	<0.0001
Fasting Insulin (PU/L)	0.295	<0.0001
Uric Acid (μmol/L)	0.288	0.0001
Fasting C-peptide (ng/mL)	0.265	0.0004
Ferritin (ng/mL)	0.26	0.0006
ALP (U/L)	0.239	0.0016
Systolic BP (mmHg)	0.21	0.0058
Diastolic BP (mmHg)	0.188	0.0134
Height (m)	0.172	0.024
FPG(mmol/L)	0.161	0.0343
CRP (mg/L)	0.14	0.0669
Total Cholesterol (mmol/L)	0.107	0.1607
LDL-C (mmol/L)	0.092	0.2295
Serum Creatinine (μmol/L)	0.077	0.3158
TIBC (μmol/L)	0.048	0.5314
Age (years)	-0.013	0.8667
Serum Iron (μmol/L)	-0.07	0.3622
HDL-C (mmol/L)	-0.183	0.0164

Data represent Spearman’s rank correlation coefficients (ρ) between ultrasound attenuation (USAT) and selected clinical/metabolic parameters. P values < 0.05 were considered statistically significant. Abbreviations: BMI, body mass index; ALT, alanine aminotransferase; GGT, γ-glutamyl transpeptidase; AST, aspartate aminotransferase; TG, triglycerides; ALP, alkaline phosphatase; BP, blood pressure; FPG, fasting plasma glucose; CRP, C-reactive protein; TC, total cholesterol; LDL-C, low-density lipoprotein cholesterol; HDL-C, high-density lipoprotein cholesterol; Scr, serum creatinine; TIBC, total iron-binding capacity.

### 3.4 Hepatic steatosis detection and categorization via logistic regression analysis

#### 3.4.1 Distribution of USAT values across different QCT categories

**Figure 4:**
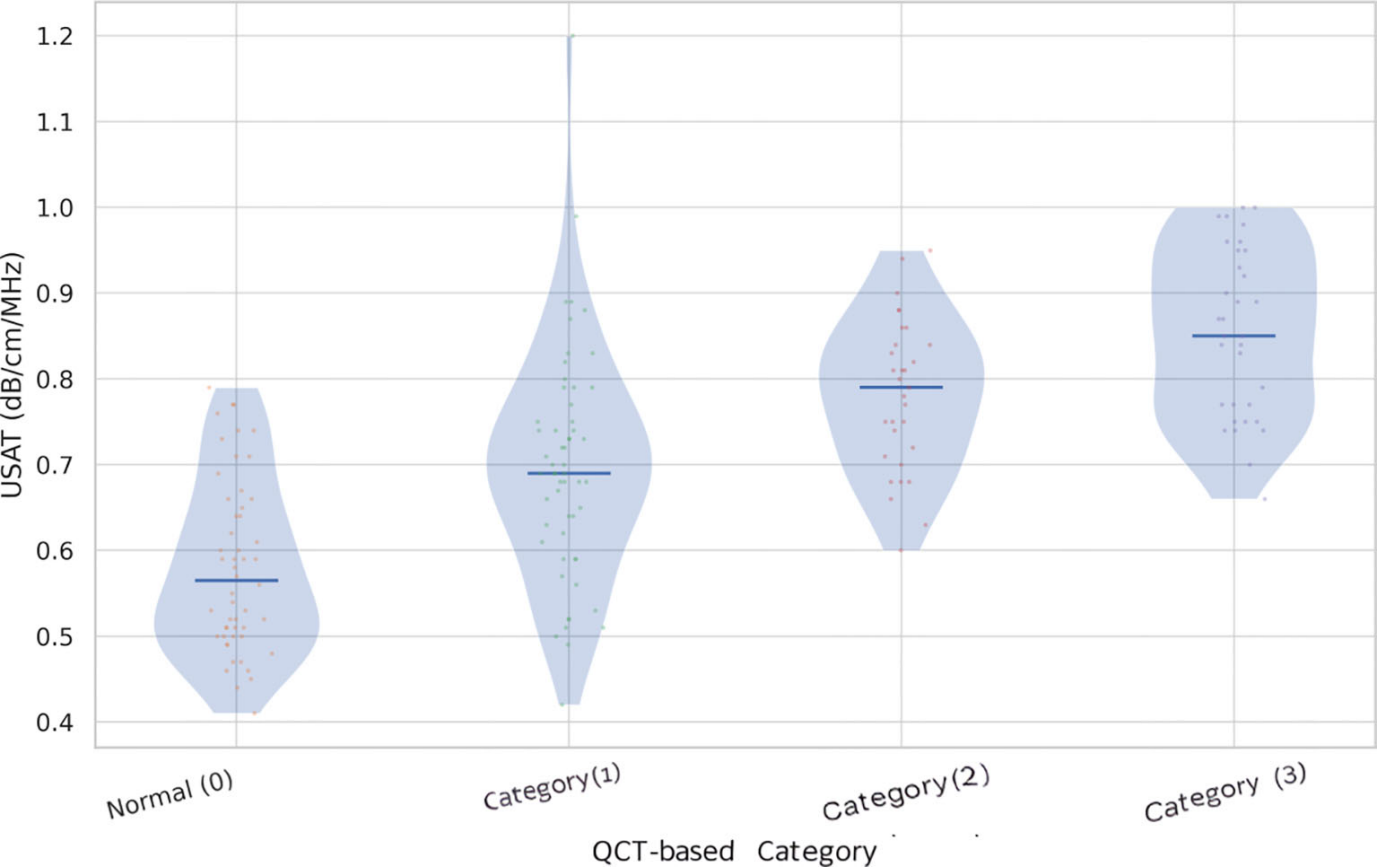
Distribution of USAT across QCT-based hepatic fat fraction categories. Violin plots show the distribution of USAT values across QCT-derived hepatic fat fraction categories. Jittered dots represent individual participants, and horizontal bars denote group medians.

To visually demonstrate the variation of USAT across different categories of fatty liver, we analyzed the raw USAT measurements across different QCT categories. Participants were divided into defined hepatic fat fraction categories according to QCT-based hepatic fat fraction categories, which was defined using hepatic fat fraction thresholds as described in the Methods section. USAT values exhibited a clear increasing trend in parallel with the hepatic fat fraction category (**Figure 4**, **Table 4**).

**Table 4:** Distribution of USAT values across different QCT categories with two decimal precision.

QCT_category	Count	Mean	STD	50%	25%	75%	Min	Max
Normal	52	0.58	0.1	0.56	0.5	0.65	0.41	0.79
Category 1	56	0.7	0.14	0.69	0.62	0.76	0.42	1.2
Category 2	31	0.78	0.09	0.79	0.72	0.84	0.6	0.95
Category 3	33	0.85	0.1	0.85	0.77	0.95	0.66	1

The table provides detailed descriptive statistics, including count, mean, standard deviation, median (50%), first quartile (25%), third quartile (75%), minimum, and maximum values, for each QCT category: Normal, Category 1, Category 2, Category 3. The USAT values show a progressive increase, indicating higher liver fat accumulation with the hepatic fat fraction category.

In the Normal group, the median USAT value was 0.565 (Interquartile Range: 0.50–0.65), indicating a relatively low liver fat content. The Category 1 group showed an increased median USAT of 0.690 (IQR: 0.62–0.76). As the degree of hepatic steatosis increased, the median USAT further rose to 0.790 (IQR: 0.72–0.84) in the Category 2 group, and reached 0.850 (IQR: 0.77–0.95) in the Category 3 group, with the interquartile range shifting to the right.

**Figure 5:**
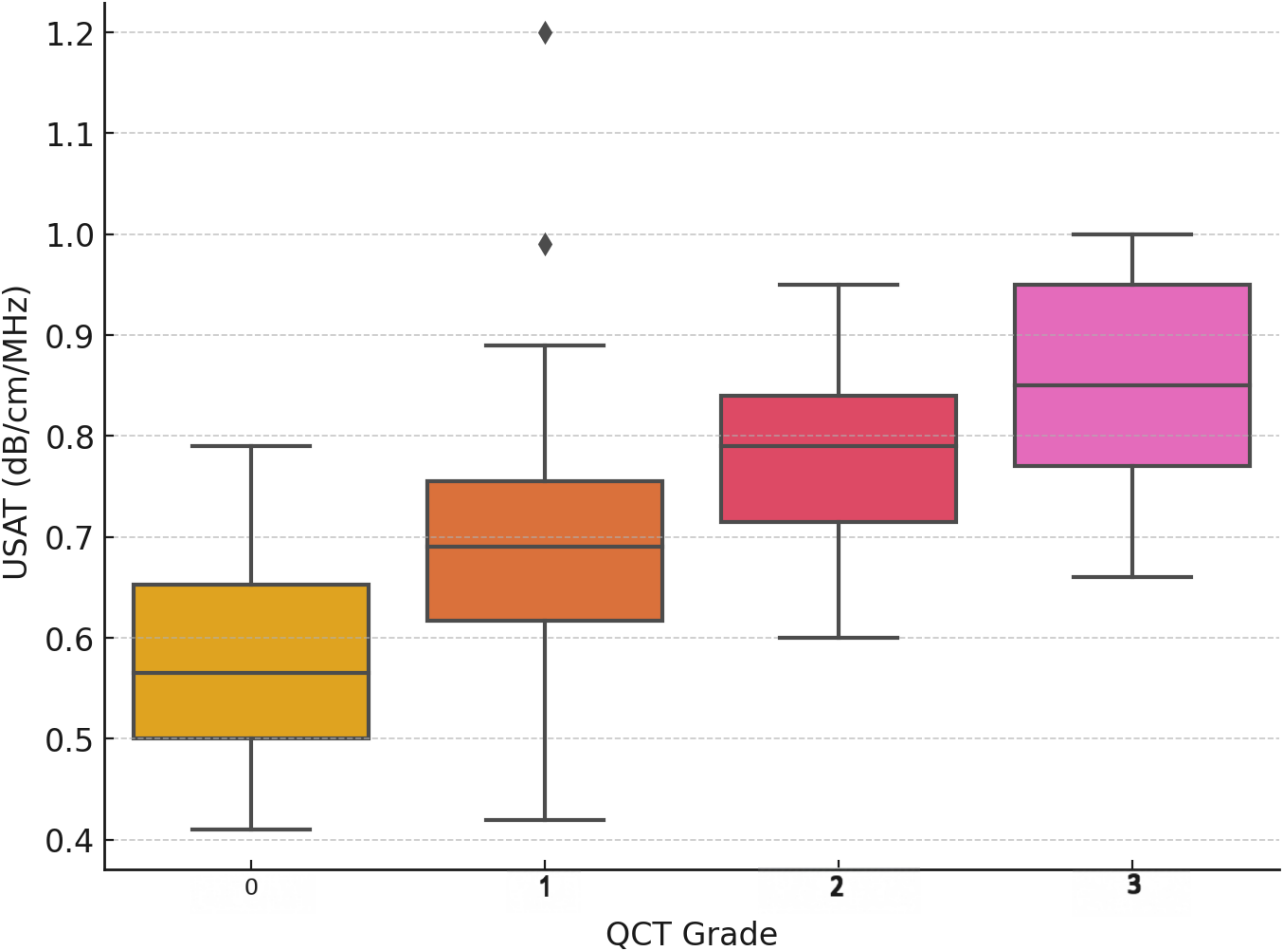
Boxplot showing the distribution of USAT values across different QCT categories. The USAT (Ultrasound Attenuation) Box plots show the distribution of USAT values across QCT-derived hepatic fat fraction categories. Individual data points are overlaid because of the modest subgroup sizes.

These findings suggest that USAT values generally increased with QCT-derived hepatic fat fraction category, supporting an overall association between USAT and hepatic fat burden. However, distributional overlap among adjacent categories, especially around the intermediate range, may limit its ability to reliably separate all hepatic fat fraction categories. This distribution trend is further visualized in the boxplot (**Figure 5**), which shows a stepwise shift in USAT values across QCT-derived categories but also demonstrates overlap between adjacent groups. Therefore, USAT may be more suitable for QCT-referenced detection of steatosis than for reliable multiclass categorization, particularly in the intermediate range.

#### 3.4.2 Prediction of MASLD presence using USAT and laboratory parameters

To compare the QCT-referenced classification ability across domains, we first developed a univariable logistic regression model based solely on USAT, using QCT category ≥1 as the comparator definition of imaging-defined steatosis. Based on out-of-fold predictions from five-fold stratified cross-validation, the USAT-only model achieved an AUC of 0.847 (95% CI, 0.780–0.902), with a sensitivity of 80.8%, specificity of 78.8%, accuracy of 80.2%, F1 score of 0.851, and Brier score of 0.140.

**Figure 6:**
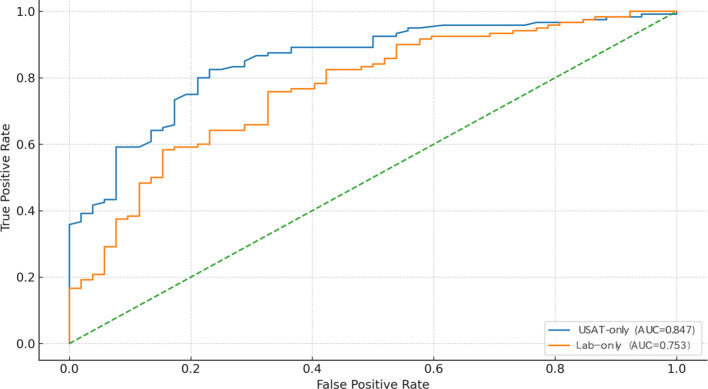
ROC curves for USAT-only and laboratory-only models for QCT-referenced detection of imaging-defined steatosis. Shaded regions indicate bootstrap-derived 95% confidence envelopes based on 2,000 bootstrap replicates.

In parallel, we constructed a laboratory-only model incorporating routinely available biochemical indicators, including liver enzymes, lipid metabolism markers, renal function indices, glucose metabolism, and inflammatory/iron-related parameters. Under the same fold-separated evaluation scheme, this comprehensive laboratory model achieved an AUC of 0.753 (95% CI, 0.665–0.829), with a sensitivity of 73.3%, specificity of 73.1%, accuracy of 73.3%, F1 score of 0.793, and Brier score of 0.185. The ROC curves for the USAT-only and laboratory-only models are shown in **Figure 6**.

Harrell’s optimism-corrected bootstrap analysis showed an optimism-corrected AUC of 0.860 for the USAT-only model, 0.782 for the laboratory-only model, and 0.868 for the fixed USAT + ALT + ferritin model. These results supported the robustness of USAT as the dominant predictor, while the laboratory-only model showed greater optimism. The diagnostic performance of the binary models is summarized in **Table 5**.

**Table 5:** Diagnostic performance of binary models for QCT-referenced detection of imaging-defined steatosis.

Model	AUC (95% CI)	Sensitivity	Specificity	Accuracy	F1 score	Brier score	N
USAT-only	0.847 (0.780–0.902)	80.8%	78.8%	80.2%	0.851	0.140	172
Lab-only, all markers	0.753 (0.665–0.829)	73.3%	73.1%	73.3%	0.793	0.185	172
Fixed integrated model: USAT + ALT + ferritin	0.845 (0.772–0.906)	85.8%	78.8%	83.7%	0.880	0.134	172
Nested L1-selected integrated model	0.830 (0.755–0.897)	78.3%	82.7%	79.7%	0.843	0.143	172

Performance estimates were based on out-of-fold predictions from five-fold stratified cross-validation. The fixed integrated model was prespecified using USAT, ALT, and ferritin. For the nested L1-selected integrated model, preprocessing and feature selection were performed exclusively within each training fold. AUC confidence intervals were estimated using 2,000 bootstrap replicates. Pairwise AUC comparisons were performed using the DeLong test and paired bootstrap resampling.

Calibration was further assessed using Brier scores and calibration plots based on out-of-fold predicted probabilities. The fixed integrated model showed the lowest Brier score, followed by the USAT-only model, whereas the laboratory-only model had the highest Brier score, indicating relatively poorer calibration. These findings suggest that the addition of ALT and ferritin may modestly improve probability calibration, although it did not significantly improve discrimination compared with USAT alone. Calibration plots are provided in **Supplementary Figure 1**.

Exploratory decision-curve analysis showed broadly similar net-benefit patterns for the USAT-only and fixed integrated models across clinically plausible threshold probabilities, whereas the laboratory-only model generally yielded lower net benefit. This pattern was consistent with the comparable AUCs of the USAT-only and fixed integrated models and suggests that the addition of ALT and ferritin did not provide a clear incremental net-benefit advantage in the present internally validated dataset. The decision-curve analysis is shown in **Supplementary Figure 2**.

Calibration was assessed using Brier scores and calibration plots based on out-of-fold predicted probabilities. The fixed integrated model showed the lowest Brier score (0.134), followed by the USAT-only model (0.140), the nested L1-selected integrated model (0.143), and the laboratory-only model (0.185). These findings suggest that the addition of ALT and ferritin may modestly improve probability calibration, although it did not significantly improve AUC compared with USAT alone. Calibration plots are provided in **Supplementary Figure 1**.

##### 3.4.2.1 *Post hoc* precision, class balance, and events-per-variable assessment

The final analytic cohort included 172 participants, comprising 52 participants in Category 0, 56 in Category 1, 31 in Category 2, and 33 in Category 3. For binary QCT-referenced detection of imaging-defined steatosis, 120 participants were classified as QCT category ≥1 and 52 as QCT category 0. The 95% CI width for the USAT-only AUC was 0.122, corresponding to a half-width of 0.061. The CI width was 0.164 for the laboratory-only model, 0.134 for the fixed USAT + ALT + ferritin model, and 0.142 for the nested L1-selected integrated model. These findings indicate acceptable precision for binary steatosis detection but greater uncertainty for laboratory-only and data-selected models.

The initial laboratory-only model included 17 candidate biochemical predictors. For binary classification, the number of QCT-positive cases was 120 and the number of QCT-negative cases was 52, corresponding to approximately 7.1 events per candidate predictor when considering QCT-positive cases and 3.1 non-events per predictor when considering the smaller outcome group. Therefore, the all-marker laboratory-only model was retained only as an exploratory benchmark. In contrast, the fixed integrated model included three prespecified predictors, namely USAT, ALT, and ferritin, corresponding to 40.0 QCT-positive cases and 17.3 QCT-negative cases per predictor. This reduced model was therefore considered more appropriate for interpretation in the present sample.

In contrast, the fixed USAT + ALT + ferritin model was prespecified and more parsimonious, with a more acceptable approximate cases-per-predictor ratio. Details are provided in **Supplementary Table 1**.

For multiclass classification, Category 2 and Category 3 included only 31 and 33 participants, respectively. With three predictors in the integrated random forest model, the approximate number of observations per predictor was 10.3 for Category 2 and 11.0 for Category 3. However, because multiclass classification requires stable separation across adjacent categories and because the Category 2 AUC remained low, the multiclass results were interpreted as exploratory rather than confirmatory.

#### 3.4.3 Ordinal logistic regression for hepatic hepatic fat fraction categories prediction

##### 3.4.3.1 Predictive value of liver function–related indicators for the hepatic fat fraction category of MASLD

**Figure 7:**
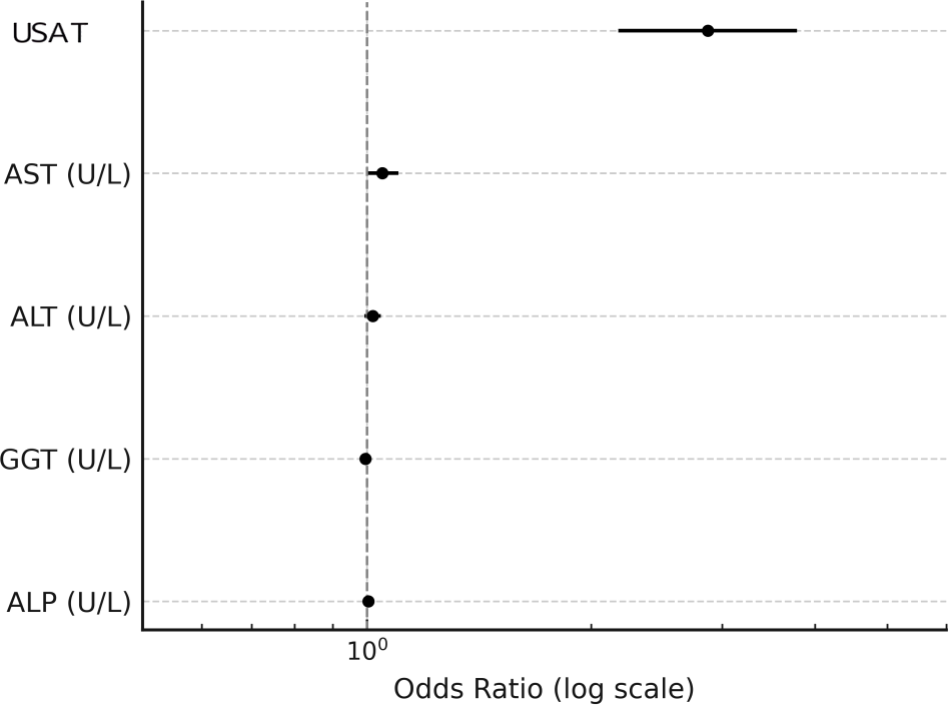
Forest plot of ordinal logistic regression for MASLD hepatic fat fraction category (QCT category 0–3). Horizontal lines indicate 95% CIs and markers indicate odds ratios (log scale). The model includes USAT(per0.1dB/cm/MHz), AST, ALT, GGT, and ALP. USAT(per0.1dB/cm/MHz) remains the only significant predictor after adjustment (OR, 2.87; 95% CI, 2.18–3.78; P <.001), while liver enzymes do not reach statistical significance.

To assess the independent predictive value of the USAT parameter in the context of conventional liver function tests, we constructed an ordinal logistic regression model including four commonly used liver enzymes—AST, ALT, GGT, and ALP—together with USAT (standardized per 0.1 dB/cm/MHz increment). The dependent variable was hepatic hepatic fat fraction categories by QCT from 0 to 3, based on predefined hepatic fat fraction thresholds (category 0: <5%, category 1: 5% to <10%, category 2: 10% to <25%, and category 3: ≥25%). As shown in **Figure 7** and **Table 6**, USAT was the only variable that remained highly significant in the model (OR=2.87, 95% CI: 2.18–3.78, P < 0.001), indicating that for every 0.1-unit increase in USAT, the likelihood of being classified into a higher QCT category nearly tripled. This finding suggests that USAT may serve as a noninvasive, quantitative imaging marker for liver fat content in hepatic fat fraction category.

**Table 6:** Ordinal logistic regression analysis of USAT and liver enzymes for MASLD hepatic fat fraction category (QCT category 0–3).

Variable	OR	95% CI (Lower)	95% CI (Upper)	P-value
USAT(per0.1dB/cm/MHz)	2.869	2.177	3.783	<0.001
AST (U/L)	1.049	0.998	1.103	0.062
ALT (U/L)	1.018	0.992	1.044	0.174
GGT (U/L)	0.996	0.987	1.005	0.427
ALP (U/L)	1.004	0.991	1.017	0.564

Data are presented as odds ratios (OR) with 95% confidence intervals (CI). MASLD hepatic fat fraction was category using QCT. USAT was standardized as ×10 for each 0.1 dB/cm/MHz increment. P values < 0.05 were considered statistically significant. USAT was scaled so that the odds ratio represents each 0.1 dB/cm/MHz increase.

AST showed a marginally significant positive association with steatosis category (OR=1.05, P=0.062), suggesting that it may reflect mild hepatocellular stress or early necroinflammatory activity. However, ALT (OR=1.02, P=0.174), GGT (OR=1.00, P=0.427), and ALP (OR=1.00, P=0.564) did not reach statistical significance, indicating that these conventional liver enzymes may have limited sensitivity in detecting mild to moderate hepatic fat accumulation, especially in the absence of prominent inflammation or fibrosis.

In summary, this model suggests that USAT may provide useful noninvasive information for assessing MASLD hepatic fat fraction category, while traditional liver enzymes showed limited associations in this analysis.

##### 3.4.3.2 Predictive value of glucolipid metabolism–related indicators for fatty liver hepatic fat fraction category

**Figure 8:**
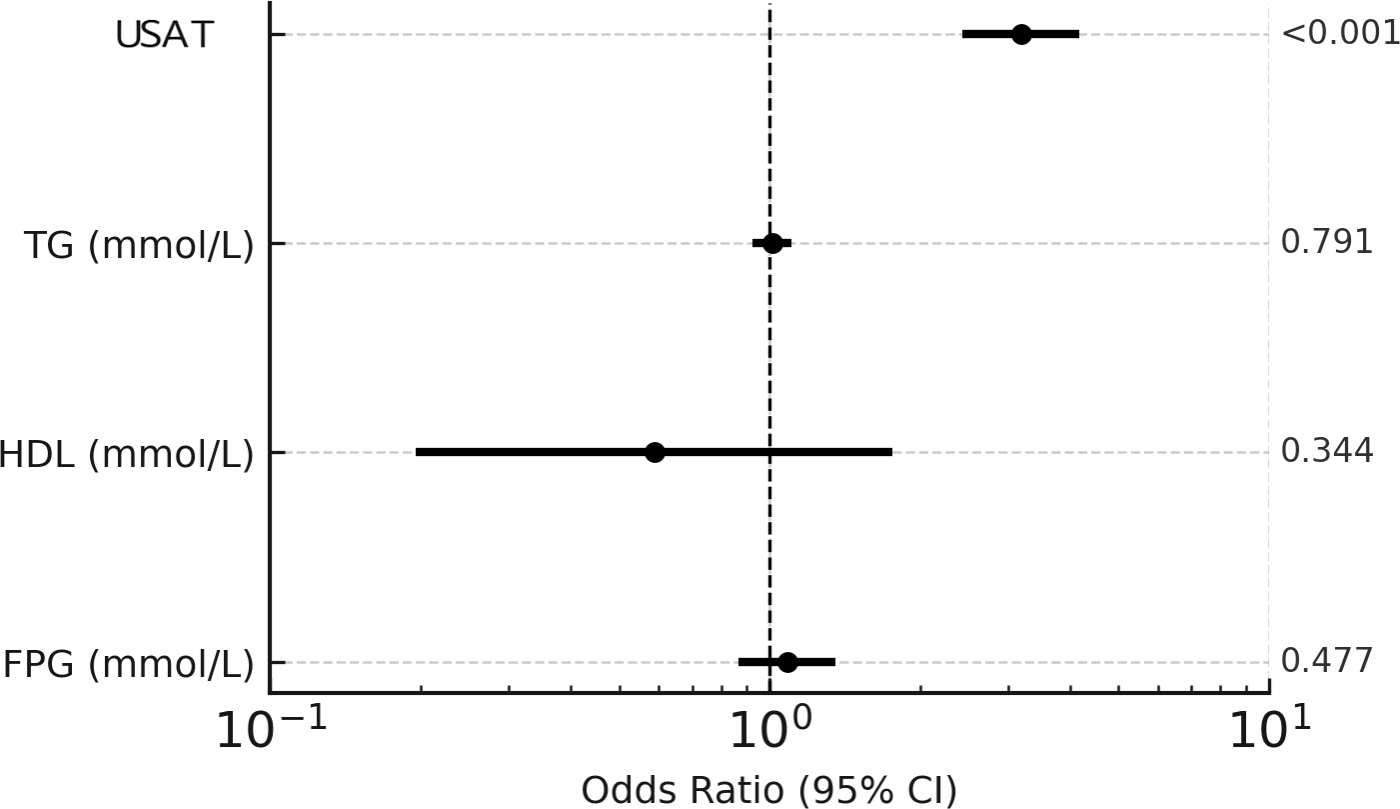
Forest plot of ordinal logistic regression for QCT-based hepatic fat fraction categories (categories 0–3): USAT×10 with metabolic covariates (TG, HDL, FPG).USAT×10 rescales USAT per 0.1 dB/cm/MHz. Dots represent odds ratios and horizontal bars represent 95% CIs; dashed line indicates OR=1. The x-axis is on a log scale.

To investigate the association between classic metabolic risk factors and hepatic fat fraction category, an ordinal logistic regression model was constructed including triglyceride (TG) level, high-density lipoprotein (HDL) level, fasting plasma glucose (FPG) level, and standardized USAT (per 0.1 dB/cm/MHz increment) as independent variables. The dependent variable was QCT-based fatty liver category (0–3). As shown in **Figure 8** and **Table 7**, USAT was the only significant predictor (odds ratio [OR], 3.19; 95% confidence interval [CI]: 2.43, 4.18; P <.001), suggesting a consistent association with hepatic hepatic fat fraction categories after adjustment for metabolic factors. In contrast, TG (OR, 1.01; P=.791), HDL (OR, 0.59; P=.344), and FPG (OR, 1.08; P=.477) were not statistically significant.

**Table 7:** Ordinal logistic regression for QCT-based hepatic fat fraction categories (categories 0–3): associations of USAT×10 and metabolic indicators (OR, 95% CI).

Variable	OR	95% CI (Lower)	95% CI (Upper)	P-value
USAT(per0.1dB/cm/MHz)	3.187	2.43	4.179	<0.001
TG (mmol/L)	1.012	0.925	1.108	0.791
HDL (mmol/L)	0.588	0.196	1.766	0.344
FPG (mmol/L)	1.084	0.868	1.354	0.477

Model: Ordered logistic regression (logit link) with QCT category 0–3 as the outcome.USAT×10: USAT multiplied by 10 to reflect effect per 0.1 dB/cm/MHz.ORs and 95% CIs are Wald-based; two-sided P values. Covariate units: TG, HDL, FPG in mmol/L; OR per one-unit increase.

This suggests that although metabolic dysregulation is a major pathogenic mechanism of MASLD, these markers have limited predictive value for hepatic fat fraction category in the early stage or in individuals without marked metabolic abnormalities. These findings further support the central role of USAT in predicting fatty liver category, particularly for early screening in MASLD populations without typical metabolic abnormalities.

Despite TG and HDL showing p-values greater than 0.05, their biological relevance to MASLD remains significant. TG reflects impaired lipid metabolism and insulin resistance, while HDL is an important marker for lipid transport. However, in the multivariable regression model, their contribution was attenuated, and they did not remain statistically significant. These findings highlight the importance of considering both biological mechanisms and statistical significance in model selection, while suggesting that USAT remains associated with fatty liver hepatic fat fraction category in this model.

##### 3.4.3.3 Predictive value of renal and inflammatory markers for hepatic fat fraction categories

**Figure 9:**
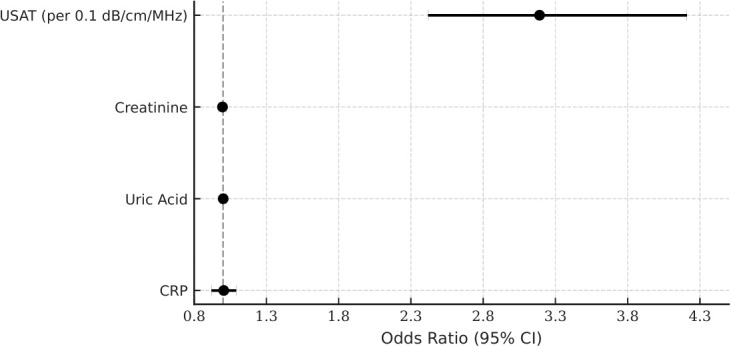
Forest plot of ordinal logistic regression for QCT-defined hepatic fat fraction categories (renal & inflammation model). Odds ratios (squares) and 95% confidence intervals (thick horizontal lines) are shown on a log scale. The dashed vertical line marks the null (OR = 1). USAT is scaled per 0.1 dB/cm/MHz (×10). Abbreviations: CRP, C-reactive protein.

To evaluate whether renal function and low-category systemic inflammation confound the association between USAT and hepatic hepatic fat fraction categories, we fitted a proportional-odds ordinal logistic regression using QCT categories 0–3 as the outcome and USAT scaled per 0.1 dB/cm/MHz (i.e., ×10), creatinine, uric acid, and CRP as predictors. As shown in **Figure 9** and **Table 8**, USAT emerged as the only statistically significant predictor (OR=3.19, 95% CI: 2.42–4.21, P < 0.001),indicating that each 0.1 dB/cm/MHz increase in USAT is associated with approximately a 3.19-fold increase in the odds of being classified into a more severe QCT category. Creatinine (OR=0.996, P=0.752), uric acid (OR=1.001, P=0.659), and CRP (OR=1.005, P=0.943) were not significant, suggesting that USAT retains robust and independent prognostic value for steatosis categorization irrespective of renal excretory variation or low-category inflammation, thereby supporting its clinical utility as a stable imaging biomarker.

**Table 8:** Ordinal logistic regression for QCT-defined hepatic fat fraction categories (renal & inflammation model).

Variable	Odds ratio	95% CI (Lower)	95% CI (Upper)	P value
USAT(per0.1dB/cm/MHz)	3.192	2.421	4.21	<0.001
Creatinine (μmol/L)	0.996	0.972	1.021	0.752
Uric acid (μmol/L)	1.001	0.997	1.004	0.659
CRP (mg/L)	1.005	0.872	1.159	0.943

Values are odds ratios with 95% confidence intervals and P values. USAT is scaled per 0.1 dB/cm/MHz (×10).

##### 3.4.3.4 Predictive value of iron metabolism markers for hepatic fat fraction categories

**Figure 10:**
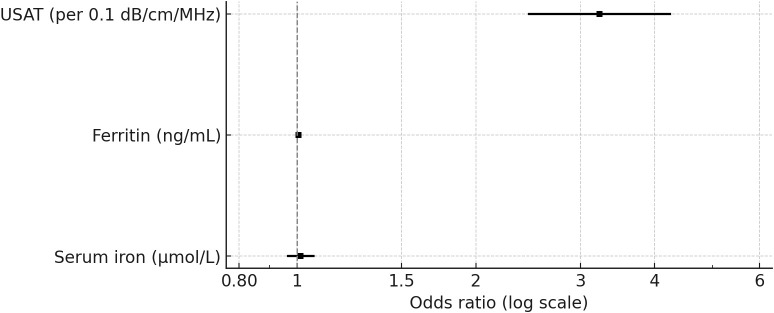
Forest plot of ordinal logistic regression for QCT-based hepatic fat fraction categories (predictors: USAT [per 0.1 dB/cm/MHz], ferritin, and serum iron). Squares indicate odds ratios; horizontal lines indicate 95% CIs; vertical dashed line marks OR=1.

**Figure 11:**
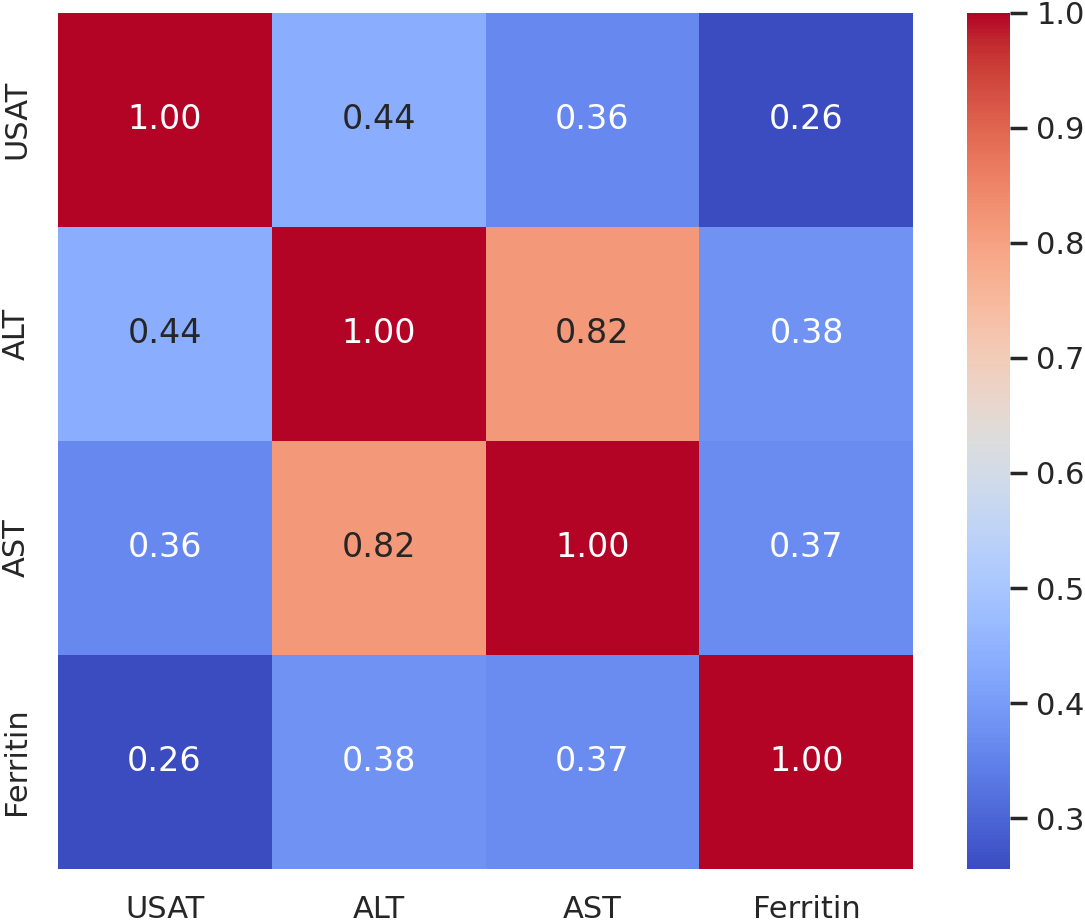
correlation matrix among candidate liver injury and iron metabolism markers, including USAT, ALT, AST, and ferritin. ALT and AST were highly correlated (r = 0.83), while USAT and ferritin showed weak correlation with hepatic enzymes.

To examine whether iron metabolism relates to hepatic hepatic fat fraction categories, we fitted an ordinal logistic regression using QCT categories 0–3 as the ordered outcome and included USAT scaled as ×10 (i.e., per 0.1 dB/cm/MHz), ferritin, and serum iron as predictors. As shown in **Figure 10** and **Table 9**, USAT remained a significant predictor (OR, 3.230; 95% CI, 2.459–4.242; P <.001), while ferritin was also statistically significant (OR, 1.006; 95% CI, 1.002–1.010; P=.001), suggesting that ferritin levels are associated with MASLD hepatic fat fraction category. Serum iron was not significant (OR, 1.015; 95% CI, 0.966–1.067; P=.558). Results were numerically identical across two independent fits within machine precision, supporting robustness. The correlation matrix among USAT, ALT, AST, and ferritin is shown in **Figure 11**. Overall, ferritin serves as a complementary biomarker for categorization, whereas USAT(per0.1dB/cm/MHz) remains the most stable imaging-based predictor.

**Table 9:** Ordinal logistic regression (logit link) for QCT category (0–3) with USAT(per0.1dB/cm/MHz), ferritin, and serum iron.

Variable	Odds ratio	95% CI (Lower)	95% CI (Upper)	P value
USAT (per 0.1 dB/cm/MHz)	3.229	2.445	4.263	<0.001
Ferritin (ng/mL)	1.006	1.002	1.01	0.001
Serum iron (μmol/L)	1.015	0.966	1.067	0.558

Reported are odds ratios with 95% CIs and P values from Wald tests.

Because ferritin is a nonspecific acute-phase reactant, its association with QCT-derived hepatic fat fraction categories should be interpreted cautiously. In the present cohort, CRP showed only a weak and statistically nonsignificant correlation with USAT *ρ*=0.140, *P*=0.0669, but CRP-based stratified analyses were not sufficiently powered.

##### 3.4.3.5 Optimized integrated model analysis

To further evaluate the optimized predictive performance of combining USAT with routine biochemical markers for hepatic fat fraction categories, we developed an integrated ordinal logistic regression model incorporating USAT, ALT, ferritin, BMI, and waist-to-hip ratio. BMI and waist-to-hip ratio were included to assess whether USAT retained an independent association with QCT-derived hepatic fat fraction after adjustment for adiposity-related anthropometric factors. In the BMI and waist-to-hip ratio-adjusted integrated ordinal logistic model, USAT remained independently associated with higher QCT-derived hepatic fat fraction category (OR=2.738 per 0.1 dB/cm/MHz; 95% CI, 2.027–3.697; P < 0.001). ALT (OR=1.026; 95% CI, 1.009–1.043; P=0.003) and ferritin (OR=1.005; 95% CI, 1.001–1.008; P=0.016) also remained independently associated with QCT category, whereas BMI and waist-to-hip ratio were not statistically significant.

Although TG showed an association in univariable analysis, it was not an independent predictor in the multivariable model (P=0.791). As shown in **Table 10**, Multicollinearity analysis showed that all variables in the BMI- and waist-to-hip ratio-adjusted integrated model had VIF values below 5, including USAT *VIF*=1.55, ALT *VIF*=1.38, ferritin *VIF*=1.20, BMI *VIF*=1.30, and waist-to-hip ratio *VIF*=1.00, suggesting no severe multicollinearity among the included predictors.

**Table 10:** Variance inflation factors for the BMI- and waist-to-hip ratio-adjusted integrated ordinal logistic regression model.

Variable	VIF
USAT	1.55
ALT	1.38
Ferritin	1.20
BMI	1.30
Waist-to-hip ratio	1.00

VIF, variance inflation factor. All VIF values were below 5, indicating no severe multicollinearity among predictors in the adjusted integrated model.

In conclusion, USAT remained independently associated with QCT-derived hepatic fat fraction category after adjustment for BMI and waist-to-hip ratio, suggesting that its association with hepatic fat fraction was not fully explained by general or central adiposity. However, because the model was internally validated only and lacks external validation, its clinical implementation potential should be interpreted cautiously.

##### 3.4.3.6 Multinomial logistic regression for category-specific interpretation

To provide category-specific interpretability, a multinomial logistic regression model was fitted using QCT category 0 as the reference category. The model included USAT, ALT, ferritin, BMI, and waist-to-hip ratio. USAT showed progressively stronger associations with higher QCT-derived hepatic fat fraction categories. As shown in **Table 11**, Compared with Category 0, the odds ratios for USAT were 1.927 for Category 1, 3.685 for Category 2, and 6.833 for Category 3 per 0.1 dB/cm/MHz increase. ALT and ferritin were significantly associated only with Category 3 versus Category 0, whereas waist-to-hip ratio was not statistically significant in any category-specific comparison. Waist-to-hip ratio was not statistically significant and showed wide confidence intervals. These findings support the dominant and graded association between USAT and QCT-derived hepatic fat fraction categories, while indicating that biochemical markers mainly provided adjunctive information for more advanced categories.

**Table 11:** Multinomial logistic regression analysis using QCT category 0 as the reference category.

Comparison	Variable	Coefficient	OR (95% CI)	P value
Category 1 vs Category 0	USAT, per 0.1 dB/cm/MHz	0.656	1.927 (1.224–3.032)	0.005
Category 1 vs Category 0	ALT	0.010	1.010 (0.974–1.049)	0.584
Category 1 vs Category 0	Ferritin	0.005	1.005 (0.999–1.011)	0.074
Category 1 vs Category 0	BMI	0.210	1.234 (1.048–1.454)	0.012
Category 1 vs Category 0	Waist-to-hip ratio	1.551	4.715 (0.011–1964.4)	0.614
Category 2 vs Category 0	USAT, per 0.1 dB/cm/MHz	1.304	3.685 (2.056–6.603)	<0.001
Category 2 vs Category 0	ALT	0.028	1.028 (0.988–1.070)	0.172
Category 2 vs Category 0	Ferritin	0.006	1.006 (0.999–1.013)	0.118
Category 2 vs Category 0	BMI	0.213	1.237 (1.037–1.477)	0.018
Category 2 vs Category 0	Waist-to-hip ratio	-0.777	0.460 (0.000–500.0)	0.828
Category 3 vs Category 0	USAT, per 0.1 dB/cm/MHz	1.922	6.833 (3.462–13.487)	<0.001
Category 3 vs Category 0	ALT	0.044	1.045 (1.003–1.089)	0.033
Category 3 vs Category 0	Ferritin	0.009	1.009 (1.001–1.017)	0.029
Category 3 vs Category 0	BMI	0.190	1.210 (1.006–1.455)	0.043
Category 3 vs Category 0	Waist-to-hip ratio	-1.284	0.277 (0.000–356.6)	0.725

QCT category 0 was used as the reference category. USAT was scaled per 0.1 dB/cm/MHz increase. OR, odds ratio; CI, confidence interval; BMI, body mass index.

In the nested feature-selection analysis, USAT was selected in all five outer folds, ferritin in four folds, GGT in four folds, iron in two folds, and ALT in one fold. The nested L1-selected integrated model achieved an AUC of 0.830 (95% CI, 0.755–0.897), with sensitivity of 78.3%, specificity of 82.7%, accuracy of 79.7%, F1 score of 0.843, and Brier score of 0.143. These results indicate that USAT was the most stable predictor across resampling iterations, whereas the selection of individual biochemical markers was less stable.

#### 3.4.4 Comparative analysis of classification performance for fatty liver hepatic fat fraction category: USAT versus combined model

##### 3.4.4.1 USAT univariate model analysis

To evaluate the exploratory multiclass performance of USAT among participants with QCT-defined steatosis, a three-class random forest model was developed using USAT as the sole input variable. The dependent variable was the QCT-derived hepatic fat fraction category among Category 1, Category 2, and Category 3. Strict five-fold stratified cross-validation was applied. In each fold, the model was trained only on the training subset, and the held-out validation fold was used exclusively for performance assessment. Accuracy, macro-averaged F1 score, macro-AUC, and class-specific AUCs were calculated from pooled out-of-fold predictions.

**Figure 12:**
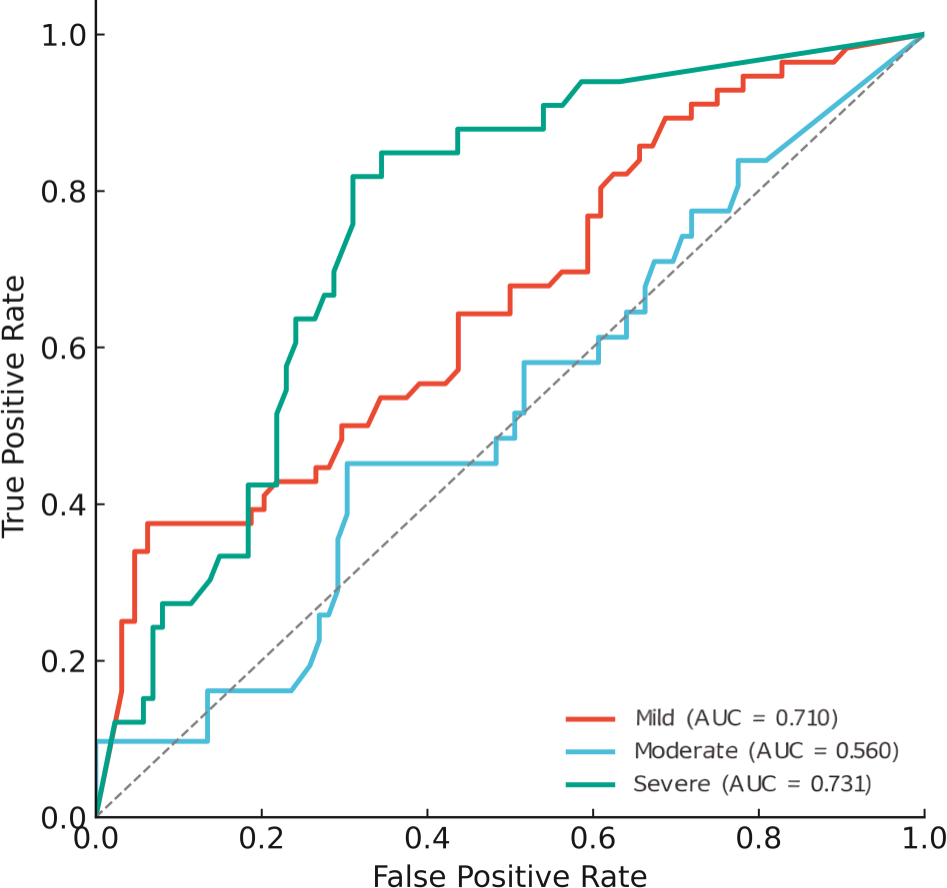
Multiclass ROC curves of the USAT-only random forest model for QCT-referenced hepatic fat fraction categorization. ROC curves were generated from pooled out-of-fold predictions under strict five-fold stratified cross-validation. Category-specific AUCs were 0.710 for Category 1, 0.560 for Category 2, and 0.731 for Category 3.

After strict fold-separated evaluation, the three-class USAT-only random forest model achieved an overall accuracy of 47.5%, an F1-macro score of 0.457, and a macro-AUC of 0.667. Class-specific AUCs were 0.710 for Category 1, 0.560 for Category 2, and 0.731 for Category 3 (**Figure 12**), indicating limited multiclass discriminative performance, particularly for the intermediate Category 2 group. These estimates reflect validation-fold performance rather than training-set performance and therefore provide a more conservative assessment of multiclass discrimination.

In summary, while USAT shows some ability to distinguish Category 3 cases, its standalone performance for multiclass hepatic fat fraction categorization is limited, particularly for moderate disease.

##### 3.4.4.2 Discriminative ability of the integrated model (USAT + biochemistry)

Building on the foundation established in Section 4.3.5, we further evaluated an exploratory three-class random forest model integrating USAT with ALT and ferritin to determine whether biochemical markers provided incremental information for QCT-referenced hepatic fat fraction categorization.

**Figure 13:**
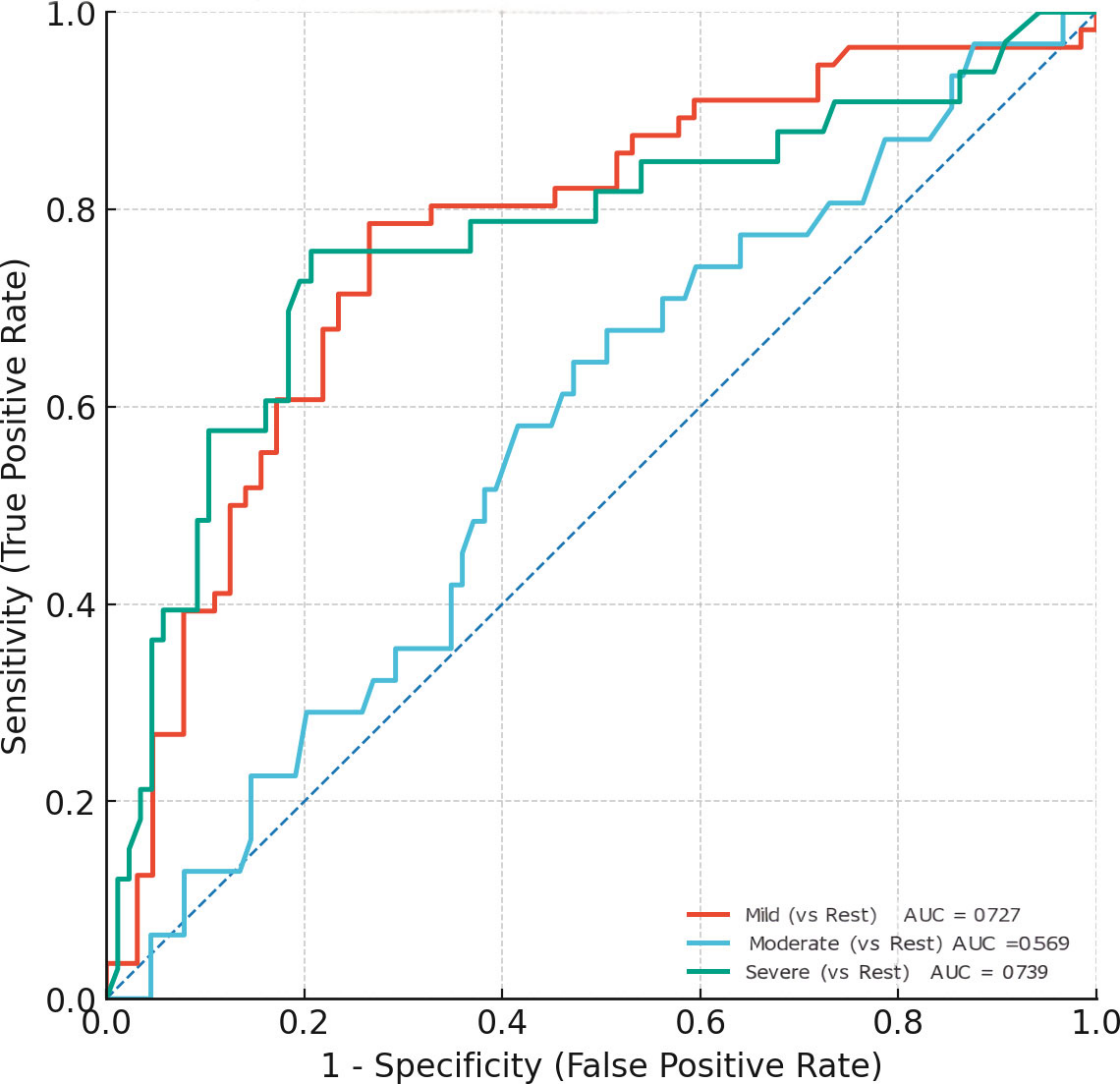
Multiclass ROC curves of the integrated random forest model combining USAT, ALT, and ferritin for QCT-referenced hepatic fat fraction categorization. ROC curves were generated from pooled out-of-fold predictions under strict five-fold stratified cross-validation. Category-specific AUCs were 0.727 for Category 1, 0.569 for Category 2, and 0.739 for Category 3.

After strict fold-separated evaluation, the three-class integrated random forest model using USAT, ALT, and ferritin achieved an overall accuracy of 52.5%, an F1-macro score of 0.482, and a macro-AUC of 0.679. Class-specific AUCs were 0.727 for Category 1, 0.569 for Category 2, and 0.739 for Category 3 (**Figure 13**).Compared with the USAT-only random forest model, the integrated model showed only modest improvement in accuracy and macro-AUC. These results suggest that biochemical markers may provide complementary information, but the improvement was limited and should be interpreted cautiously because of small class sizes and overlap between adjacent QCT-derived categories. In particular, the limited sample sizes of Category 2 *n*=31 and Category 3 *n*=33 reduced the stability of multiclass classification and increased the uncertainty of class-specific performance estimates.

Analysis of the ROC results revealed that the integrated model showed moderate discrimination for Category 1 *AUC*=0.727 and Category 3 *AUC*=0.739. However, the Category 2 group showed weak discrimination *AUC*=0.569, indicating that the model had difficulty separating the clinically important intermediate hepatic fat fraction range from adjacent categories. This limitation likely reflects substantial overlap in USAT and biochemical feature distributions between Category 2 and adjacent categories. Therefore, Category 2 performance should be interpreted as weak rather than as evidence of reliable intermediate-stage classification.

The Category 2 group remained difficult to distinguish from adjacent categories. In the corrected three-class random forest analysis, the Category 2 AUC was 0.569 for the USAT + ALT + ferritin model and 0.577 after adding TG, indicating near-random to weak discrimination at the clinically important intermediate range.

In summary, incorporating biochemical markers alongside USAT produced only modest improvement in multiclass QCT-referenced categorization. Overall performance remained limited, especially for Category 2. Therefore, the current model should not be considered reliable for intermediate hepatic fat fraction categorization and does not provide a basis for clinical decision-support model development. The corrected random forest performance metrics are summarized in **Table 12**.

**Table 12:** Corrected random forest performance using strict fold-separated evaluation.

Model	Accuracy	F1-macro	Macro-AUC	Category 1 AUC	Category 2 AUC	Category 3 AUC	Evaluation
USAT-only random forest	47.5%	0.457	0.667	0.710	0.560	0.731	Five-fold out-of-fold
USAT + ALT + ferritin random forest	52.5%	0.482	0.679	0.727	0.569	0.739	Five-fold out-of-fold
USAT + ALT + TG + ferritin random forest, sensitivity analysis	55.8%	0.514	0.694	0.752	0.577	0.752	Five-fold out-of-fold

Random forest models were evaluated using strict five-fold stratified cross-validation. Model fitting was performed only in the training subset of each fold, and performance metrics were calculated from pooled out-of-fold predictions. Macro-AUC was calculated as the mean of one-vs-rest AUCs across Category 1, Category 2, and Category 3. Category 0 was excluded from this three-class analysis because binary steatosis detection was evaluated separately.

#### 3.4.5 Subgroup analysis

##### 3.4.5.1 Gender subgroup analysis

**Figure 14:**
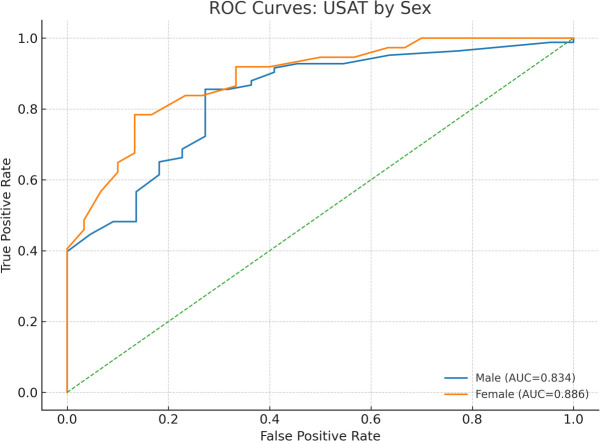
ROC curves demonstrate that USAT shows better diagnostic performance in the female group compared to the male group, with AUCs of 0.886 and 0.834, respectively.

To investigate potential gender-related differences in the diagnostic performance of USAT for fatty liver, a subgroup analysis was conducted according to sex. The male group consisted of 105 participants (normal/fatty liver = 22/83), while the female group included 67 participants (normal/fatty liver = 30/37). Binary logistic regression models were constructed using USAT alone (normal vs fatty liver), and ROC curves were generated to determine AUC values. As shown in **Table 13**, USAT achieved an AUC of 0.886 in females, which was slightly higher than the 0.834 observed in males. The ROC curve for the female subgroup consistently lay above that of the male subgroup (**Figure 14**), suggesting that, for a given USAT value, females had a higher probability of being correctly classified as having fatty liver disease. Potential explanations for this difference include sex-related variations in fat distribution, metabolic characteristics, and patterns of biochemical parameter changes.

**Table 13:** Diagnostic performance of USAT by gender.

Gender	Sample size	AUC	Normal vs MASLD	95% CI
Male	105	0.834	22/83	0.732–0.918
Female	67	0.886	30/37	0.805–0.953

AUC calculated from a univariate logistic regression model using USAT, with fatty liver defined as QCT ≥ 1.

##### 3.4.5.2 Age subgroup analysis

Participants were stratified into three age groups (20–40 years, 40–60 years, and ≥60 years) to assess the diagnostic performance of USAT across age strata.

**Figure 15:**
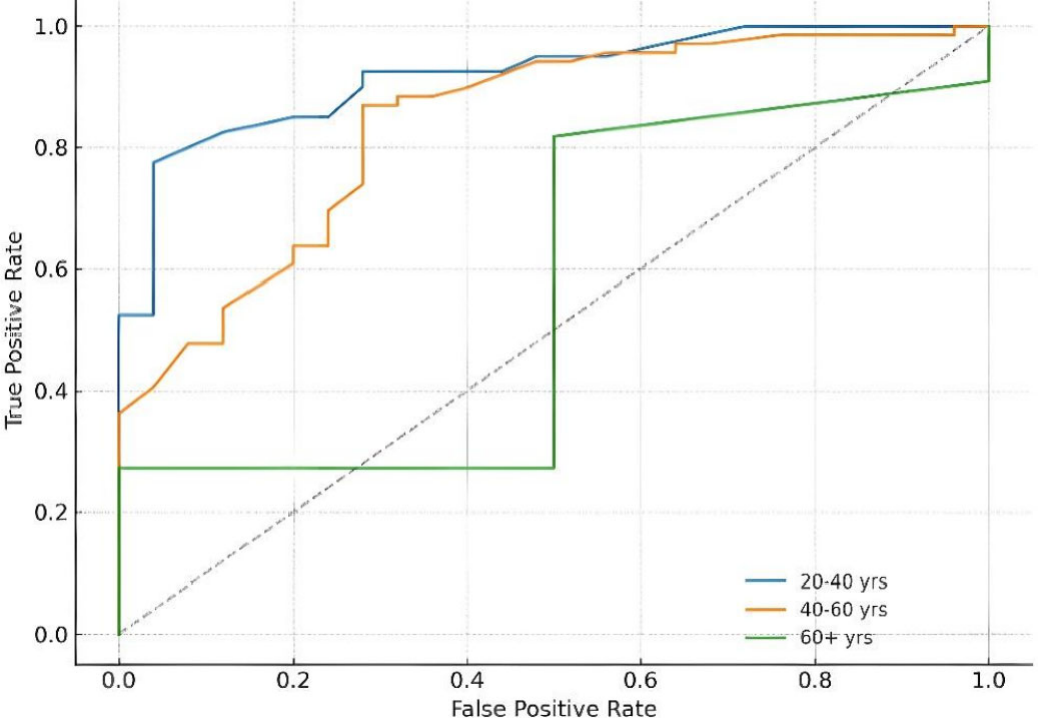
ROC Curves of USAT by Age Group.ROC curves illustrating the diagnostic performance of USAT for fatty liver across three age groups. The 20–40 years group achieved the highest AUC, while the ≥60 years group had the lowest.

The AUC for detecting hepatic steatosis was 0.842 (95% CI: 0.730–0.913) in the 20–40-year group (n = 44), 0.906 (95% CI: 0.842–0.957) in the 40–60-year group (n = 97), and 0.717 (95% CI: 0.545–0.861) in the ≥60-year group (n = 31) (**Table 14**; **Figure 15**).

**Table 14:** Diagnostic performance of USAT by age group.

Age group	Sample size (n)	Number of normal cases	Number of MASLD	AUC
20–40	44	13	31	0.842
40–60	97	28	69	0.906
≥60	31	11	20	0.717

AUC values represent the area under the ROC curve for the USAT-only model in diagnosing fatty liver across different age groups; higher values indicate better diagnostic performance.

Although the 40–60-year group showed a numerically higher AUC, no formal statistical comparison between age subgroups was performed; therefore, these findings should be interpreted with caution.

##### 3.4.5.3 BMI subgroup analysis

Participants were categorized into three BMI subgroups according to the “Guidelines for the Prevention and Control of Overweight and Obesity in Chinese Adults (WS/T 428-2013)”: normal weight (BMI < 24 kg/m²), overweight (24 ≤ BMI < 28 kg/m²), and obese (BMI ≥ 28 kg/m²). The diagnostic performance of USAT for detecting MASLD (QCT category ≥1) was evaluated within each subgroup.

**Figure 16:**
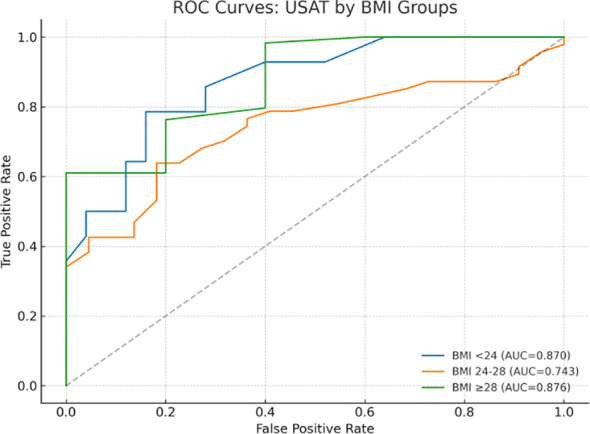
ROC curves of USAT by BMI category curves illustrating the diagnostic performance of USAT for fatty liver across different BMI categories. ROC curves illustrate the diagnostic performance of USAT for fatty liver across BMI categories. AUC estimates varied across subgroups and should be interpreted cautiously because of limited subgroup sample sizes.

As shown in **Table 15** and **Figure 16**, USAT achieved an AUC of 0.870 (95% CI: 0.739–0.968, n=61) in the normal weight group, 0.743 (95% CI: 0.627–0.851, n=57) in the overweight group, and 0.876 (95% CI: 0.672–1.000, n=54) in the obese group. These values correspond to the respective numbers of normal and MASLD cases within each subgroup.

**Table 15:** Diagnostic performance of USAT by BMI category.

BMI category	Sample size (n)	Normal cases	MASLD cases	AUC	95% CI
Normal weight (<24)	61	28	33	0.870	0.739–0.968
Overweight (24–28)	57	15	42	0.743	0.627–0.851
Obese (≥28)	54	9	45	0.876	0.672–1.000

AUC values represent the area under the ROC curve for **Table 13**. the USAT-only model in diagnosing fatty liver across different BMI categories. Higher values indicate better diagnostic performance. BMI classification is based on the “Guidelines for the Prevention and Control of Overweight and Obesity in Chinese Adults (WS/T 428-2013)”.

USAT performance varied across BMI subgroups, with numerically higher AUCs in the normal-weight and obese groups than in the overweight group. Given the differences in subgroup sizes and the limited number of normal cases in certain groups, these findings should be interpreted cautiously. Given the differences in subgroup sizes and the limited number of normal cases in certain groups, these results should be interpreted with caution.

##### 3.4.5.4 Summary of subgroup analysis

The subgroup analyses suggested that USAT performance varied across different population characteristics. Overall, higher AUC values were recorded in females, middle-aged individuals, and overweight participants, suggesting greater sensitivity of USAT in these groups. The observed sex differences may be linked to hormonal influences and patterns of fat distribution, while the superior performance in middle-aged adults could be associated with more pronounced imaging features of fatty liver. The higher AUC in overweight individuals may reflect the enhanced detectability of hepatic fat deposition at earlier disease stages. Although the AUC was slightly lower in the obese group, it remained within a clinically acceptable range, indicating that USAT retains diagnostic value across various BMI categories.

It should be noted that attenuation coefficient-based measurements may be affected by the thickness of subcutaneous adipose tissue. As this parameter was not assessed in the present study, its potential impact should be explored in future studies.

In conclusion, subgroup findings should be interpreted cautiously because of limited subgroup sizes and the absence of formal interaction testing. These analyses do not establish broad applicability across diverse clinical contexts.

## 4 Discussion

This study used QCT-derived hepatic fat fraction categories as pragmatic imaging comparator labels and systematically evaluated the QCT-referenced performance of USAT alone and in combination with metabolic biomarkers. Our findings are generally in line with previous studies showing that attenuation coefficient–based ultrasound techniques can reflect hepatic fat burden and provide useful noninvasive information for steatosis assessment. Prior studies on ultrasound attenuation analysis or related attenuation-based parameters have similarly reported increasing attenuation values with increasing hepatic fat fraction categories, supporting the biological plausibility of the present results. In this context, our study adds further evidence by evaluating USAT within a QCT-referenced framework and by exploring whether biochemical markers may provide incremental value beyond imaging alone. QCT is a non-invasive and quantifiable imaging technique that can estimate hepatic fat content in health examination settings. In this study, QCT-derived hepatic fat fraction categories provided operational comparator labels for model development; however, these labels should be interpreted as study-specific stratification categories rather than as a validated clinical grading system.

Although QCT provides a quantitative and accessible estimate of hepatic fat content in health examination settings, histology and MRI-PDFF remain the more widely accepted reference methods for hepatic fat quantification. Therefore, the present findings should be interpreted as QCT-referenced method-comparison results rather than definitive diagnostic accuracy against a gold standard.

In the present study, USAT values increased progressively with QCT-defined hepatic fat fraction categories, supporting its quantitative association with hepatic fat accumulation. This pattern is broadly consistent with previous reports on attenuation-based ultrasound techniques, which have also shown stepwise increases across hepatic fat fraction categories and good performance for detecting the presence of steatosis. At the same time, our analysis showed that discrimination of the intermediate Category 2 group remained weak. In the corrected random forest analysis, the Category 2 AUC was 0.560 for the USAT-only model and 0.569 for the USAT + ALT + ferritin model, indicating near-random to weak separation. This is a clinically important limitation because the 10–25% hepatic fat fraction range represents a key intermediate decision boundary. Therefore, the current results support QCT-referenced steatosis detection more strongly than reliable multiclass hepatic fat fraction categorization (Jiang et al., 2023). Discrimination was relatively better at the lower and higher ends of the spectrum, whereas the intermediate Category 2 group showed substantial overlap with adjacent categories.

We also evaluated a laboratory-only baseline model using routinely available biochemical indicators. Its performance was lower than that of the USAT-only model, suggesting that blood-based markers alone may reflect the metabolic background of steatotic liver disease but are less directly linked to hepatic fat content itself. This interpretation is broadly consistent with previous clinical observations that aminotransferases, lipid-related indices, and inflammatory or iron-related biomarkers may be associated with MASLD, yet their diagnostic performance is often variable and insufficient when used in isolation. By comparison, USAT provides a direct quantitative signal related to tissue attenuation and therefore may be more closely aligned with imaging-defined steatosis burden. For QCT-referenced detection of imaging-defined steatosis, the laboratory-only model achieved an AUC of 0.753 (95% CI, 0.665–0.829), with 73.3% sensitivity and 73.1% specificity, whereas the USAT-only model yielded an AUC of 0.847 (95% CI, 0.780–0.902), with 80.8% sensitivity and 78.8% specificity. USAT-only significantly outperformed the laboratory-only model, with an AUC difference of 0.094 (DeLong P = 0.015; paired bootstrap P = 0.018). The fixed integrated model including USAT, ALT, and ferritin achieved an AUC of 0.845 (95% CI, 0.772–0.906), but did not significantly improve AUC compared with USAT alone. Calibration analysis suggested a modest improvement in probability calibration for the fixed integrated model, as reflected by a slightly lower Brier score. However, exploratory decision-curve analysis did not demonstrate a clear incremental net-benefit advantage over the USAT-only model. Therefore, although ALT and ferritin may provide adjunctive biological information, the present data do not support a strong claim of superior clinical utility for the integrated model.

In the liver function subgroup, AST showed a marginal association in the liver enzyme model, whereas ALT was retained in the adjusted integrated model together with USAT and ferritin. This choice reflects the clinical relevance of ALT as a marker of hepatocellular injury in early steatosis. After adjustment for BMI and waist-to-hip ratio, ALT and ferritin remained independently associated with QCT-derived hepatic fat fraction category, supporting their adjunctive biological value. However, their addition did not significantly improve AUC compared with USAT alone, indicating that these biomarkers should be interpreted as complementary markers rather than as evidence of superior discrimination (Kumada et al., 2022; Wang et al., 2024).

Because USAT was correlated with BMI, body weight, and waist-to-hip ratio, potential confounding by adiposity was specifically evaluated. In the adjusted ordinal logistic regression model including BMI and waist-to-hip ratio, USAT remained independently associated with QCT-derived hepatic fat fraction category. This finding suggests that the USAT signal was not solely attributable to general or central adiposity. Nevertheless, residual confounding cannot be excluded, particularly because waist circumference was not available as a complete standalone variable and alcohol intake, hepatosteatogenic medication exposure, and menopausal status were not fully available for all participants.

To improve categorization performance, we further combined USAT with selected biochemical markers. The integrated model showed only modest improvement over USAT alone in multiclass classification. Although biochemical markers may provide complementary clinical information, the corrected fold-separated results do not support a claim of markedly improved discrimination. The random forest analyses were re-evaluated using strict fold-separated validation because multiclass models are particularly vulnerable to optimistic bias when evaluated on training data or when preprocessing is not confined to training folds. After re-analysis using pooled out-of-fold predictions, the corrected performance estimates were substantially more conservative than the initial estimates. The integrated random forest model achieved an accuracy of 52.5%, F1-macro of 0.482, and macro-AUC of 0.679, indicating limited multiclass discrimination rather than strong classification performance. These findings support the interpretation that the multiclass random forest analysis is exploratory and should not be used as evidence of clinical readiness. This finding is in line with the general concept reported in previous imaging studies that combining quantitative imaging parameters with clinical or laboratory variables may improve risk stratification by capturing both tissue-level changes and the underlying metabolic milieu. In our cohort, the added value of ALT, and ferritin suggests that imaging and metabolic markers provide partly complementary information rather than representing redundant domains alone. Although TG showed the strongest correlation within the glucose–lipid metabolism group in our preliminary subgroup analysis, it was not retained in the final integrated model. This discrepancy highlights the distinction between univariable correlation and multivariable modeling. While TG reflects lipid export and insulin resistance, its predictive contribution overlapped substantially with USAT and liver enzymes, leading to attenuation of independent effect in the multivariable framework. Despite TG and HDL having p - values greater than 0.05, their biological relevance to MASLD remains significant. TG reflects impaired lipid metabolism and insulin resistance, while HDL is an important marker for lipid transport. However, in the multivariable regression model, their contribution was attenuated, possibly due to collinearity with other variables or the absence of pronounced metabolic abnormalities in the study population (Pirmoazen et al., 2020).

USAT, by directly measuring the attenuation of ultrasound waves through liver tissue, primarily reflects hepatic fat content and is associated with changes in fat accumulation. In contrast, TG and HDL are part of the broader metabolic context and did not consistently reach statistical significance in the multivariable models. These findings suggest that USAT may provide complementary noninvasive information in the evaluation of hepatic steatosis.

ALT and ferritin provided complementary biological information related to hepatocellular injury and iron metabolism, but their addition did not significantly improve AUC compared with USAT alone. This finding underscores the importance of model-based selection beyond correlation ranking, and suggests that TG remains mechanistically relevant but not indispensable once imaging and iron-related markers are incorporated.

From a mechanistic perspective, ferritin played a particularly important role. As the primary intracellular iron storage protein, ferritin levels reflect both iron metabolism and chronic inflammatory status (Giacomazzi et al., 2022). The “iron overload–fat deposition” hypothesis posits that excess free iron generates hydroxyl radicals via the Fenton reaction, inducing lipid peroxidation and mitochondrial injury, ultimately leading to hepatocellular dysfunction and lipid accumulation. This pathway is recognized as a critical mechanism in the progression of MASLD to MASH (Ayres et al., 2024).

The association between ferritin and hepatic fat fraction categories observed in our study is partly consistent with previous reports showing that ferritin may be elevated in patients with metabolic dysfunction and fatty liver. However, this association should be interpreted cautiously, as ferritin is also influenced by systemic inflammation and other metabolic conditions. Similarly, triglyceride-related abnormalities have been widely linked to insulin resistance and hepatic lipid dysregulation in MASLD. In our data, however, TG did not remain an independent predictor in the final multivariable framework, suggesting that its contribution may be partly captured by other imaging or biochemical variables. Although fasting C-peptide, an indirect marker of β-cell function, was not included in the final model, the associations of ALT and ferritin with QCT-derived hepatic fat fraction categories support the relevance of hepatocellular injury and iron/inflammatory metabolic burden in fatty liver pathogenesis. The combined model may help describe complementary biological domains, but the present results do not demonstrate clear superiority for multiclass categorization.

The sample-size and class-imbalance limitations are particularly relevant to the multiclass analyses. Although the overall cohort included 172 participants, only 31 and 33 participants were available in Category 2 and Category 3, respectively. This limited the stability of multinomial classification and likely contributed to the poor discrimination of Category 2. Therefore, the multiclass random forest results should be regarded as exploratory. The binary QCT-referenced detection analysis was more stable, particularly for the parsimonious USAT-only and fixed USAT + ALT + ferritin models, whereas the all-marker laboratory-only model was retained only as an exploratory benchmark because of the relatively low events-per-variable ratio.

Subgroup analysis revealed that USAT performed better in females, middle-aged individuals, and the obese group participants compared to males, younger individuals, and overweight participants. These differences may be explained by hormonal influences, fat distribution patterns, and variations in hepatic fat deposition.

Conventional scoring tools such as the fibrosis-4 index (FIB-4) and NAFLD fibrosis score (NFS) are primarily designed for fibrosis risk assessment rather than steatosis detection (Kumada et al., 2022). In contrast, the present approach focuses on the detection and categorization of hepatic steatosis using quantitative USAT measurements combined with routine biochemical markers. The potential clinical applicability of this approach remains uncertain and requires external validation and formal clinical-utility assessment.

A formal head-to-head comparison with established noninvasive scoring systems was not performed because several required components were unavailable or incomplete in the present dataset. For example, FLI requires waist circumference, HSI requires diabetes status, NAFLD-LFS requires detailed metabolic syndrome and insulin-resistance variables, and FibroScan-AST requires vibration-controlled transient elastography parameters, which were not collected in this cohort. Future studies should prospectively collect these variables to compare USAT-based models with established noninvasive scores and to evaluate incremental value.

The generalizability of these findings is limited by the study setting and eligibility criteria. The present cohort was recruited from a single Chinese tertiary-care hospital and consisted of adults undergoing health examination or clinical evaluation who were able to complete both USAT and QCT. In addition, participants with several major comorbid conditions or exposures were excluded, including severe cardiovascular disease, hepatic malignancy or cirrhosis, known malignancy, autoimmune disorders, endocrine diseases, pregnancy or lactation, and recent use of medications that may alter liver function. Therefore, the present results are most applicable to relatively stable Chinese adults evaluated in similar tertiary health-examination settings. They should not be directly generalized to community-based populations, other ethnic groups, patients with advanced liver disease or major systemic comorbidities, medication-exposed populations, or primary-care settings without further validation.

Although ultrasound attenuation and routine biochemical tests are potentially accessible tools, the present data do not establish their performance in grassroots, rural, or primary-care settings. The study was conducted in a tertiary-care hospital with standardized imaging procedures and selected participants who were able to undergo both USAT and QCT. Therefore, application to lower-resource or community settings should be regarded as a future research direction rather than a current clinical recommendation (Caussy et al., 2018; Pappa and Wenzl, 2023; Dejeu et al., 2024).

The combined USAT + ALT + ferritin model should be viewed as an exploratory internally validated model rather than a deployable clinical tool. Although the required measurements are potentially available in routine health examination settings, implementation in primary care or population screening should not be recommended without external validation, assessment across different devices and operators, and formal clinical-utility evidence. At present, this strategy should be considered hypothesis-generating and suitable for further validation studies, rather than as a basis for screening, clinical risk stratification, individualized intervention, or longitudinal monitoring.

In addition, although the adjusted model included BMI and waist-to-hip ratio to account for adiposity-related confounding, waist circumference as a standalone variable was not available. Information on alcohol intake, hepatosteatogenic medication exposure, including statins, metformin, tamoxifen, and corticosteroids, and menopausal status was not complete for all participants. Therefore, these variables could not be formally incorporated into the main multivariable models, and residual confounding remains possible.

Limitations of this study include several issues related to sample size, validation, and generalizability. First, the sample size was relatively modest and class imbalance was present, particularly for Category 2 and Category 3, which included only 31 and 33 participants, respectively. This limited the stability of multinomial classification, increased the uncertainty of class-specific AUC estimates, and reduced the reliability of data-driven predictor selection. No *a priori* sample-size calculation was performed. Although we added *post hoc* precision analysis, EPV assessment, and sensitivity analyses using simpler prespecified models, the multiclass findings should be interpreted as exploratory and require validation in larger cohorts. In particular, the corrected random forest results based on strict fold-separated evaluation showed limited accuracy and macro-AUC, indicating that the multiclass machine-learning results should be considered hypothesis-generating only.

Second, generalizability is limited because this was a single-center study conducted in a Chinese tertiary-care hospital. The cohort consisted of adults undergoing health examination or clinical evaluation who were able to complete both USAT and QCT. Substantial exclusions were applied, including severe cardiovascular disease, cirrhosis, hepatocellular carcinoma, known malignancy, autoimmune disorders, endocrine diseases, pregnancy or lactation, and recent medication exposure that could alter liver function. Therefore, the findings may apply only to relatively stable Chinese adults in similar tertiary health-examination settings and should not be directly generalized to community-based populations, primary-care populations, non-Chinese or multiethnic populations, patients with advanced liver disease or major systemic comorbidities, or medication-exposed populations. In addition, all USAT examinations were performed by a single experienced operator using a standardized protocol; therefore, inter-operator reproducibility could not be assessed. Although five valid measurements were obtained for each participant and the median value was used to reduce within-examination variability, formal intra-operator reproducibility analysis using repeated examinations and intraclass correlation coefficients was not performed. Although USAT, QCT, and laboratory testing were performed within a short assessment window, exact hour-level intervals between USAT and QCT were not available for all participants. Acute illness and major procedures were excluded, but short-term changes related to fasting status, weight fluctuation, or transient illness cannot be completely ruled out.

Another important limitation is that QCT was used as a pragmatic imaging comparator rather than a histological or MRI-PDFF reference standard. Although QCT has been reported to correlate with MRI-PDFF, discordance may occur among QCT, MRI-PDFF, and histological steatosis grading, particularly around clinically relevant lower thresholds such as 5%. In addition, because individual-level paired continuous QCT-derived hepatic fat fraction and USAT-derived fat fraction values were not available for formal method-agreement analysis, Bland–Altman analysis, intraclass correlation coefficients, and limits-of-agreement analysis could not be robustly estimated in the present study. Future studies should validate USAT against MRI-PDFF or histology and include paired continuous quantitative measurements to allow formal agreement analysis.

## 5 Conclusion

Based on QCT-derived hepatic fat fraction categories, this study evaluated USAT alone and in combination with routine biochemical parameters for QCT-referenced detection and preliminary categorization of hepatic steatosis in a single-center Chinese tertiary-care cohort. USAT showed acceptable performance for detecting imaging-defined steatosis, whereas multiclass hepatic fat fraction categorization remained limited, particularly for the intermediate Category 2 group. The addition of ALT and ferritin did not significantly improve AUC over USAT alone, although it modestly improved sensitivity, F1 score, and calibration-related performance.

These findings should be interpreted as internally validated, QCT-referenced, and exploratory. They may be most relevant to relatively stable Chinese adults undergoing health examination or clinical evaluation in similar tertiary-care settings. The findings should not be directly generalized to community-based populations, primary-care populations, non-Chinese or multiethnic populations, patients with advanced liver disease, major cardiovascular disease, malignancy, autoimmune disease, pregnancy or lactation, or individuals exposed to medications that may affect hepatic fat content or liver biochemistry. External multicenter validation, preferably against MRI-PDFF or histology and across different ultrasound platforms, is required before the approach can be considered for clinical screening, disease stratification, or longitudinal follow-up.

## Data Availability

The original contributions presented in the study are included in the article/**Supplementary Material**. Further inquiries can be directed to the corresponding authors.
