# Supplementary material for: Integrated model based on ultrasound attenuation and metabolic biomarkers for noninvasive assessment of hepatic fat fraction categories in MASLD: a QCT-referenced study
Source: Front Physiol. 2026 May 29;17:1804061. doi: 10.3389/fphys.2026.1804061 (PMC13259794; doi:10.3389/fphys.2026.1804061)
Supplement: Supplementary file 1 [file SupplementaryFile1.docx]

**Completed STARD 2015 Checklist**

Checklist for reporting diagnostic accuracy studies; completed where applicable for a QCT-referenced detection/model-comparison study

*Manuscript title: Integrated Model Based on Ultrasound Attenuation and Metabolic Biomarkers for Noninvasive Assessment of Hepatic Fat Fraction Categories in MASLD: A QCT-Referenced Study*

STARD 2015 is completed where applicable because the manuscript includes QCT-referenced detection analyses. QCT is treated as a pragmatic imaging comparator rather than a histological or MRI-PDFF gold standard.

| **Item** | **Section/topic** | **Checklist item** | **Status** | **Location in manuscript** | **Notes** |
| --- | --- | --- | --- | --- | --- |
| 1 | Title or abstract | Identify the article as a study of diagnostic accuracy using at least one measure of accuracy. | Reported | Title; Abstract | The article uses QCT-referenced performance and AUC/sensitivity/specificity; wording now avoids gold-standard diagnostic accuracy claims. |
| 2 | Abstract | Structured summary of study design, methods, results, and conclusions. | Reported | Abstract | Structured Background, Methods, Results, Conclusion provided. |
| 3 | Introduction | Scientific and clinical background, including intended use and clinical role of index test. | Reported | Introduction | MASLD burden, imaging context, USAT rationale, and intended exploratory role are described. |
| 4 | Introduction | Study objectives and hypotheses. | Reported | Introduction | Objectives define QCT-referenced assessment of USAT/integrated model for detection and preliminary categorization. |
| 5 | Methods: Study design | Whether data collection was planned before index and reference standard were performed. | Reported | Materials and Methods: Study Design and Participants | Prospective, single-center, cross-sectional design reported. |
| 6 | Methods: Participants | Eligibility criteria. | Reported | Materials and Methods: Inclusion and Exclusion Criteria | Inclusion/exclusion criteria listed. |
| 7 | Methods: Participants | Basis for identifying potentially eligible participants. | Reported | Materials and Methods: Study Design and Participants | Adults undergoing liver QCT for clinical or screening purposes were consecutively enrolled. |
| 8 | Methods: Participants | Where and when participants were identified. | Reported | Materials and Methods: Study Design and Participants | First Hospital of Jilin University, September 2023 to March 2025. |
| 9 | Methods: Participants | Whether participants formed a consecutive, random, or convenience series. | Reported | Materials and Methods: Study Design and Participants | Consecutive enrollment stated. |
| 10a | Methods: Test methods | Index test details sufficient for replication. | Reported | Materials and Methods: Ultrasound Attenuation Imaging (USAT) | USAT device, transducer, fasting, position, acoustic window, S5 ROI, valid measurements, median value, quality exclusion, blinding reported. |
| 10b | Methods: Test methods | Reference standard details sufficient for replication. | Partially reported | Materials and Methods: Quantitative Computed Tomography (QCT) | Scanner, software, ROI placement, category thresholds and consensus reading described; detailed kVp/mA/slice thickness/phantom details require verification or limitation statement. |
| 11 | Methods: Test methods | Rationale for choosing reference standard. | Reported with qualification | Introduction; Materials and Methods: Quantitative Computed Tomography (QCT); Discussion | QCT is reframed as pragmatic imaging comparator, not a histology/MRI-PDFF gold standard. |
| 12a | Methods: Test methods | Definition and rationale for test positivity cutoffs/categories for index test. | Reported | Materials and Methods: Statistical Analysis; Results: Prediction of MASLD Presence Using USAT and Laboratory Parameters | Youden threshold for binary probability; USAT modeled continuously and scaled per 0.1 dB/cm/MHz. |
| 12b | Methods: Test methods | Definition and rationale for reference standard cutoffs/categories. | Reported | Materials and Methods: Quantitative Computed Tomography (QCT) | QCT categories <5, 5-<10, 10-<25, >=25% defined as study-specific operational strata. |
| 13a | Methods: Test methods | Whether index test performers/readers were blinded to reference standard. | Reported | Materials and Methods: Ultrasound Attenuation Imaging (USAT) | USAT operator blinded to QCT results. |
| 13b | Methods: Test methods | Whether reference standard performers/readers were blinded to index test. | Partially reported | Materials and Methods: Quantitative Computed Tomography (QCT) | Independent radiologist review and consensus reported; explicit blinding to USAT should be confirmed if applicable. |
| 14 | Methods: Analysis | Methods for estimating or comparing diagnostic accuracy. | Reported | Materials and Methods: Statistical Analysis | AUC, sensitivity, specificity, DeLong/paired bootstrap, calibration, DCA, nested CV reported. |
| 15 | Methods: Analysis | How indeterminate test results and missing data were handled. | Reported | Materials and Methods: Ultrasound Attenuation Imaging (USAT); Statistical Analysis; Results: Participant Characteristics | Failed USAT quality measurements excluded; incomplete imaging/lab data excluded; remaining predictors imputed within training folds. |
| 16 | Methods: Analysis | Methods for analyses of variability in diagnostic accuracy, distinguishing prespecified from exploratory. | Reported | Results: Subgroup Analysis; Discussion | Sex/age/BMI subgroup analyses and multiclass RF analyses described as exploratory. |
| 17 | Methods: Analysis | Intended sample size and how it was determined. | Reported | Materials and Methods: Statistical Analysis; Results: Post hoc precision, class balance, and events-per-variable assessment | No a priori sample-size calculation; post hoc precision and EPV analyses added. |
| 18 | Results: Participants | Flow diagram of participants. | Reported | Figure 1: Participant flow diagram | STROBE/TRIPOD-style flow diagram included; 200 screened -> 172 analyzed. |
| 19 | Results: Participants | Baseline demographic and clinical characteristics. | Reported | Results: Tables 1-2 | Baseline characteristics and QCT-category comparisons are reported. |
| 20 | Results: Participants | Number of participants satisfying eligibility criteria, included, and excluded with reasons. | Reported | Results: Participant Characteristics; Figure 1 | 15 refused QCT; 13 incomplete data; 172 included. |
| 21a | Results: Participants | Distribution of severity of target condition among those with target condition. | Reported | Results: Comparison of Clinical and Biochemical Parameters Across QCT-Based Steatosis Categories; Table 2 | QCT categories and hepatic fat fraction summaries by grade reported. |
| 21b | Results: Participants | Distribution of alternative diagnoses among those without target condition. | Not applicable/Not reported | N/A | Participants without QCT-defined steatosis were Category 0; alternative diagnoses were not the focus. |
| 22 | Results: Test results | Time interval and clinical interventions between index test and reference standard. | Partially reported | Materials and Methods: Ultrasound Attenuation Imaging (USAT); Laboratory Assessments | USAT within 3 days of QCT; exact hour-level interval not available for all participants; no intervention reported. |
| 23 | Results: Test results | Cross-tabulation of index test results by reference standard. | Partially reported | Results: Distribution of USAT Values Across Different QCT Categories; Prediction of MASLD Presence Using USAT and Laboratory Parameters; Table 5 | Continuous USAT distributions by QCT category and performance metrics are reported; no binary 2x2 table at selected threshold is provided. |
| 24 | Results: Estimates | Estimates of diagnostic accuracy and precision. | Reported | Results: Table 5; Figure 6 | AUC with 95% CI, sensitivity, specificity, accuracy, F1, Brier score reported. |
| 25 | Results: Harms | Any adverse events from performing index test or reference standard. | Not reported | Not clearly stated | No adverse events were reported; consider adding explicit statement if confirmed. |
| 26 | Discussion | Study limitations, sources of bias, statistical uncertainty, and generalizability. | Reported | Discussion: Limitations | QCT comparator limits, sample size/class imbalance, missing confounders, reproducibility and generalizability addressed. |
| 27 | Discussion | Implications for practice, including intended use and clinical role. | Reported with caution | Discussion; Conclusion | Clinical deployment not recommended; future validation required. |
| 28 | Other information | Registration number and registry. | Not reported | Not clearly stated | Prospective trial/observational registration status should be disclosed per editorial comment E-7. |
| 29 | Other information | Where full study protocol can be accessed. | Not reported | Not clearly stated | Protocol availability should be stated if applicable. |
| 30 | Other information | Sources of funding and role of funders. | Reported | Title page: Funding | Funding listed; role of funders should be specified if required by journal. |

Note: Locations are reported using manuscript section headings rather than page numbers, because page numbers may change during production. Items marked as Not applicable or Partially reported reflect the QCT-referenced and internally validated nature of the study.
