# Supplementary material for: Integrated model based on ultrasound attenuation and metabolic biomarkers for noninvasive assessment of hepatic fat fraction categories in MASLD: a QCT-referenced study
Source: Front Physiol. 2026 May 29;17:1804061. doi: 10.3389/fphys.2026.1804061 (PMC13259794; doi:10.3389/fphys.2026.1804061)
Supplement: Supplementary file 2 [file SupplementaryFile2.docx]

**Completed TRIPOD+AI Checklist**

Checklist for reporting clinical prediction model development/evaluation studies using regression or machine-learning methods

*Manuscript title: Integrated Model Based on Ultrasound Attenuation and Metabolic Biomarkers for Noninvasive Assessment of Hepatic Fat Fraction Categories in MASLD: A QCT-Referenced Study*

This checklist is completed for a QCT-referenced prediction-model and method-comparison study. D = development; E = evaluation. The study uses regression models and exploratory random forest analyses; it is internally validated and not proposed for clinical deployment.

| **Item** | **Section/topic** | **D/E** | **Checklist item** | **Status** | **Location in manuscript** | **Notes** |
| --- | --- | --- | --- | --- | --- | --- |
| 1 | Title | D;E | Identify the study as developing or evaluating a multivariable prediction model, the target population, and the outcome to be predicted. | Reported | Title page/main title; Abstract | Title identifies integrated model, USAT/metabolic biomarkers, MASLD, hepatic fat fraction categories, and QCT-referenced framework. |
| 2 | Abstract | D;E | Provide a structured summary addressing objectives, design, setting, participants, predictors, outcome, model methods, performance, and conclusions. | Reported | Abstract | Abstract reports cohort size, predictors, validation strategy, AUCs, Category 2 limitation, and external validation requirement. |
| 3a | Introduction: Background | D;E | Explain the healthcare context and rationale for developing/evaluating the prediction model, including references to existing models where relevant. | Reported | Introduction | MASLD burden, MRI-PDFF/biopsy/QCT context, USAT rationale, and need for noninvasive assessment are described. |
| 3b | Introduction: Target population and intended purpose | D;E | Describe the target population and intended purpose of the prediction model in the care pathway, including intended users. | Reported | Introduction; Materials and Methods: Study Design and Participants; Inclusion and Exclusion Criteria | Target population limited to Chinese adults undergoing health examination/clinical screening in tertiary-care settings; intended use is exploratory QCT-referenced detection/preliminary categorization. |
| 3c | Introduction: Health inequalities/fairness context | D;E | Describe any known health inequalities between sociodemographic groups in the target population, where relevant. | Partially reported | Results: Subgroup Analysis; Discussion | Sex, age, and BMI subgroup analyses are reported. Formal fairness analysis was not performed and generalizability is limited. |
| 4 | Objectives | D;E | Specify study objectives, including whether the study develops or evaluates a prediction model, or both. | Reported | Introduction | Objectives state evaluation of USAT and integrated models for QCT-referenced detection and preliminary categorization. |
| 5a | Methods: Data source | D;E | Describe data sources for model development/evaluation and rationale/representativeness. | Reported | Materials and Methods: Study Design and Participants; Inclusion and Exclusion Criteria | Prospective single-center cross-sectional cohort at First Hospital of Jilin University; representativeness and limitations discussed. |
| 5b | Methods: Study dates | D;E | Describe dates of participant recruitment and data collection. | Reported | Materials and Methods: Study Design and Participants | Recruitment from September 2023 to March 2025. |
| 6a | Methods: Setting | D;E | Specify key setting information, including type, number, and location of centers. | Reported | Materials and Methods: Study Design and Participants; Inclusion and Exclusion Criteria | Single Chinese tertiary-care hospital setting is specified. |
| 6b | Methods: Eligibility | D;E | Describe inclusion and exclusion criteria. | Reported | Materials and Methods: Inclusion and Exclusion Criteria | Inclusion and exclusion criteria are listed, including major comorbidities, medication exposure, pregnancy/lactation, and imaging/data quality. |
| 6c | Methods: Interventions | D;E | If interventions are relevant to the model, describe them and their timing. | Not applicable | N/A | No intervention was administered; observational imaging/laboratory study. |
| 7 | Methods: Data preparation | D;E | Describe data preprocessing and data-quality checks, including whether similar across groups. | Reported | Materials and Methods: Imaging Examinations; Laboratory Assessments; Statistical Analysis; Results: Participant Characteristics; Prediction of MASLD Presence Using USAT and Laboratory Parameters | USAT quality thresholds, QCT ROI rules, missing-data handling, training-fold preprocessing, and fold separation are described. |
| 8a | Methods: Outcome definition | D;E | Define the predicted outcome and time horizon; describe how and when assessed and justify outcome choice. | Reported | Materials and Methods: Quantitative Computed Tomography (QCT); Statistical Analysis | Outcomes are QCT category >=1 for binary detection and QCT-derived categories 0-3/1-3 for categorization; QCT categorized as operational comparator labels. |
| 8b | Methods: Outcome assessor qualifications | D;E | If outcome assessment requires subjective interpretation, describe assessor qualifications and characteristics where relevant. | Reported | Materials and Methods: Quantitative Computed Tomography (QCT) | QCT images were independently analyzed by experienced radiologists with third-reader consensus. |
| 8c | Methods: Outcome assessment blinding | D;E | Report blinding details for outcome assessment. | Partially reported | Materials and Methods: Ultrasound Attenuation Imaging (USAT); Quantitative Computed Tomography (QCT) | USAT operator was blinded to QCT results. QCT readings used independent/consensus review; blinding to USAT/lab data should be verified in final text if applicable. |
| 9a | Methods: Predictor selection | D | Describe candidate predictor selection and any pre-selection before modeling. | Reported | Materials and Methods: Statistical Analysis; Results: Optimized Integrated Model Analysis; Multinomial Logistic Regression for Category-Specific Interpretation | Candidate laboratory markers, USAT, prespecified fixed model, and nested L1-selected feature selection are described. |
| 9b | Methods: Predictor definition/timing | D;E | Clearly define predictors and how/when measured, including blinding details where relevant. | Reported | Materials and Methods: Ultrasound Attenuation Imaging (USAT); Laboratory Assessments | USAT acquisition, laboratory assays, timing window, and key predictors are described. |
| 9c | Methods: Predictor assessor qualifications | D;E | If predictor measurement requires subjective interpretation, describe assessor qualifications. | Reported | Materials and Methods: Ultrasound Attenuation Imaging (USAT); Quantitative Computed Tomography (QCT) | USAT operator expertise and radiologist image assessment are described. |
| 10 | Methods: Sample size | D;E | Explain how sample size was determined and justify sufficiency. | Reported | Materials and Methods: Statistical Analysis; Results: Post hoc precision, class balance, and events-per-variable assessment | No a priori sample-size calculation; post hoc precision, EPV, and class-balance assessment added. |
| 11 | Methods: Missing data | D;E | Describe missing-data handling and reasons for missingness. | Reported | Materials and Methods: Statistical Analysis; Results: Participant Characteristics; Figure 1 | Complete-case analytic cohort of 172 from 200 screened; incomplete imaging/lab data exclusion and fold-wise imputation for remaining predictors described. |
| 12a | Methods: Analysis dataset use | D | Describe how data were used for model development/evaluation, including data splitting and sample-size considerations. | Reported | Materials and Methods: Statistical Analysis | Nested five-fold stratified CV, strict fold separation, and out-of-fold predictions are described. |
| 12b | Methods: Predictor handling | D | Describe predictor functional forms, scaling, transformations, and standardization. | Reported | Materials and Methods: Statistical Analysis; Results: Ordinal Logistic Regression for Hepatic Fat Fraction Categories Prediction; Optimized Integrated Model Analysis | USAT scaled per 0.1 dB/cm/MHz; logistic/ordinal/multinomial model predictors and scaling reported. |
| 12c | Methods: Model type and development steps | D | Describe model type, rationale, modeling steps, hyperparameter tuning if any, and internal validation. | Reported | Materials and Methods: Statistical Analysis; Results: Hepatic Steatosis Detection and Categorization via Logistic Regression Analysis; Comparative Analysis of Classification Performance | Logistic regression, ordinal/multinomial regression, nested feature selection, and exploratory random forest with strict cross-validation are reported. |
| 12d | Methods: Heterogeneity/clustering | D;E | Describe methods to handle/quantify heterogeneity across groups or centers. | Partially reported | Results: Subgroup Analysis; Discussion | Single-center design; subgroup analyses by sex/age/BMI were exploratory. No clustering/multicenter heterogeneity assessment. |
| 12e | Methods: Performance measures | D;E | Describe all performance measures and plots, including discrimination, calibration, and clinical utility. | Reported | Materials and Methods: Statistical Analysis; Results: Table 5; Supplementary Figures S1-S2 | AUC, sensitivity, specificity, accuracy, F1, Brier score, calibration plots, DeLong/paired bootstrap, and exploratory DCA are reported. |
| 12f | Methods: Model updating | E | For model evaluation, describe model updating/recalibration if performed. | Not applicable | N/A | No external model evaluation or model updating was performed. |
| 12g | Methods: Prediction calculation for evaluated models | E | For model evaluation, describe how model predictions were calculated. | Partially reported | Materials and Methods: Statistical Analysis; Results: Tables 5 and 11 | Predictions obtained from fold-separated models; deployable formula/software object is not provided because no clinical deployment is claimed. |
| 13 | Methods: Class imbalance | D;E | If class imbalance exists, describe why and how it was handled and any recalibration. | Reported | Materials and Methods: Statistical Analysis; Results: Post hoc precision, class balance, and events-per-variable assessment; Comparative Analysis of Classification Performance; Discussion | Class imbalance and limited Category 2/3 sample sizes are reported; results interpreted as exploratory. |
| 14 | Methods: Fairness | D;E | Describe methods and rationale for addressing model fairness. | Partially reported | Results: Subgroup Analysis; Discussion | No formal fairness analysis; exploratory sex/age/BMI subgroup analyses and generalizability limitations are reported. |
| 15 | Methods: Model output | D | Describe prediction model output, classification thresholds, and rationale. | Reported | Materials and Methods: Statistical Analysis; Results: Table 5 | Binary predicted probabilities, Youden threshold, and multiclass predicted class/probabilities are reported. |
| 16 | Methods: Development vs evaluation dataset differences | D;E | Describe differences between development and evaluation datasets. | Not applicable | N/A | No independent external evaluation dataset; internal validation only. This limitation is reported. |
| 17 | Methods: Ethics | D;E | Name ethics committee and consent/waiver. | Reported | Title page: Compliance with Ethical Standards; Materials and Methods: Study Design and Participants | Ethics approval No. 24K183-001 and written informed consent reported. |
| 18a | Open science: Funding | D;E | Provide funding sources and funder roles. | Reported | Title page: Funding | Funding sources listed. Funder role should be verified in final submission if required. |
| 18b | Open science: Conflicts of interest | D;E | Declare conflicts and financial disclosures. | Reported | Title page: Conflict of Interest | COI statement present; Mindray disclosure should be included per reviewer comment. |
| 18c | Open science: Protocol | D;E | State where protocol can be accessed or that no protocol is available. | Partially reported | Not clearly stated | If no protocol exists, add explicit statement in manuscript or supplementary material. |
| 18d | Open science: Registration | D;E | Provide registration details or state not registered. | Partially reported | Not clearly stated | Trial/observational registration status should be disclosed per editorial comment E-7. |
| 18e | Open science: Data sharing | D;E | Provide data availability details. | Reported | Title page: Data Availability | De-identified data available from corresponding author upon reasonable request, subject to approvals. |
| 18f | Open science: Code sharing | D;E | Provide analysis code availability details. | Partially reported | Not clearly stated | Add code availability statement if possible. |
| 19 | Patient and public involvement | D;E | Describe patient/public involvement or state none. | Not reported | Not clearly stated | If none, add statement: Patients/public were not involved in design, conduct, reporting, or dissemination. |
| 20a | Results: Participant flow | D;E | Describe participant flow and number with/without outcome; flow diagram encouraged. | Reported | Results: Participant Characteristics; Figure 1 | 200 screened, 15 refused QCT, 13 incomplete data, 172 analyzed. |
| 20b | Results: Participant characteristics | D;E | Report participant characteristics, predictors, outcomes, missingness, and key demographic differences. | Reported | Results: Tables 1-2; Supplementary Table S3 | Baseline characteristics, QCT categories, and effect sizes/FDR in supplementary table. |
| 20c | Results: Comparison of development/evaluation data | E | For model evaluation, compare evaluation dataset with development data. | Not applicable | N/A | No separate external evaluation dataset. |
| 21 | Results: Number of participants/events per analysis | D;E | State number of participants and outcome events in each analysis. | Reported | Results: Participant Characteristics; Prediction of MASLD Presence Using USAT and Laboratory Parameters; Comparative Analysis of Classification Performance | Counts for QCT categories, binary outcome groups, and RF categories are reported. |
| 22 | Results: Model specification | D | Provide complete prediction model details to allow prediction/reuse, including restrictions. | Partially reported | Results: Tables 5-12; Materials and Methods: Statistical Analysis | Model variables and coefficients/ORs reported; no deployable clinical calculator provided because the model remains exploratory. |
| 23a | Results: Model performance | D;E | Report performance estimates with confidence intervals, including key subgroups where relevant. | Reported | Results: Table 5; Table 12; Subgroup Analysis tables | AUCs with 95% CI for binary models; RF metrics; subgroup AUCs. |
| 23b | Results: Heterogeneity in performance | D;E | Report heterogeneity in performance across groups if examined. | Reported | Results: Subgroup Analysis | Sex, age, and BMI subgroup analyses reported as exploratory. |
| 24 | Results: Model updating | E | Report model updating results if performed. | Not applicable | N/A | No model updating performed. |
| 25 | Discussion: Interpretation | D;E | Provide overall interpretation in context of objectives, prior studies, and fairness where relevant. | Reported | Discussion | Findings interpreted with QCT-referenced limitations, Category 2 limitation, calibration/DCA, and generalizability. |
| 26 | Discussion: Limitations | D;E | Discuss limitations, bias, statistical uncertainty, and generalizability. | Reported | Discussion: Limitations | Sample size, class imbalance, QCT comparator, lack of external validation, missing confounders, reproducibility limitations described. |
| 27a | Discussion: Input data quality in use | D | Describe how low-quality or unavailable input data should be assessed/handled when using the model. | Partially reported | Materials and Methods: Imaging Examinations; Laboratory Assessments; Statistical Analysis; Discussion | USAT quality thresholds and imaging quality requirements described; no deployable model proposed. |
| 27b | Discussion: Human interaction/expertise | D | Describe whether user interaction is required and what expertise is needed. | Reported | Materials and Methods: Ultrasound Attenuation Imaging (USAT); Discussion | USAT acquisition requires trained ultrasound operator; deployment not recommended without device/operator validation. |
| 27c | Discussion: Future research | D;E | Discuss future directions with focus on applicability and generalizability. | Reported | Discussion; Conclusion | External multicenter validation, MRI-PDFF/histology validation, device/operator validation, and larger cohorts are recommended. |

Note: Locations are reported using manuscript section headings rather than page numbers, because page numbers may change during production. Items marked as Not applicable or Partially reported reflect the QCT-referenced and internally validated nature of the study.
