# Supplementary material for: Integrated model based on ultrasound attenuation and metabolic biomarkers for noninvasive assessment of hepatic fat fraction categories in MASLD: a QCT-referenced study
Source: Front Physiol. 2026 May 29;17:1804061. doi: 10.3389/fphys.2026.1804061 (PMC13259794; doi:10.3389/fphys.2026.1804061)
Supplement: Supplementary file 3 [file SupplementaryFile3.docx]

Supplementary Figure S1. Calibration plots for binary QCT-referenced detection models.


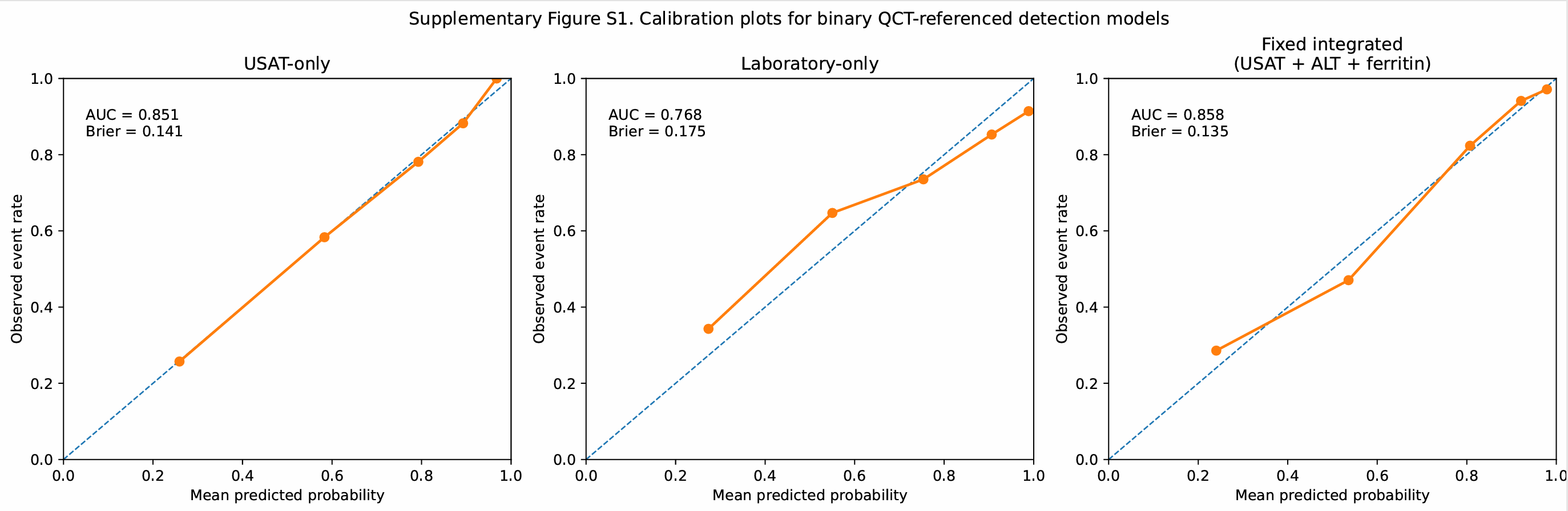


Calibration plots were generated using out-of-fold predicted probabilities from five-fold stratified cross-validation. The diagonal line represents perfect calibration. The fixed integrated model showed a slightly lower Brier score than the USAT-only model, whereas the laboratory-only model showed the highest Brier score.
