# Supplementary material for: Integrated model based on ultrasound attenuation and metabolic biomarkers for noninvasive assessment of hepatic fat fraction categories in MASLD: a QCT-referenced study
Source: Front Physiol. 2026 May 29;17:1804061. doi: 10.3389/fphys.2026.1804061 (PMC13259794; doi:10.3389/fphys.2026.1804061)
Supplement: Supplementary file 4 [file SupplementaryFile4.docx]

Supplementary Figure S2. Exploratory decision-curve analysis for binary QCT-referenced detection models.


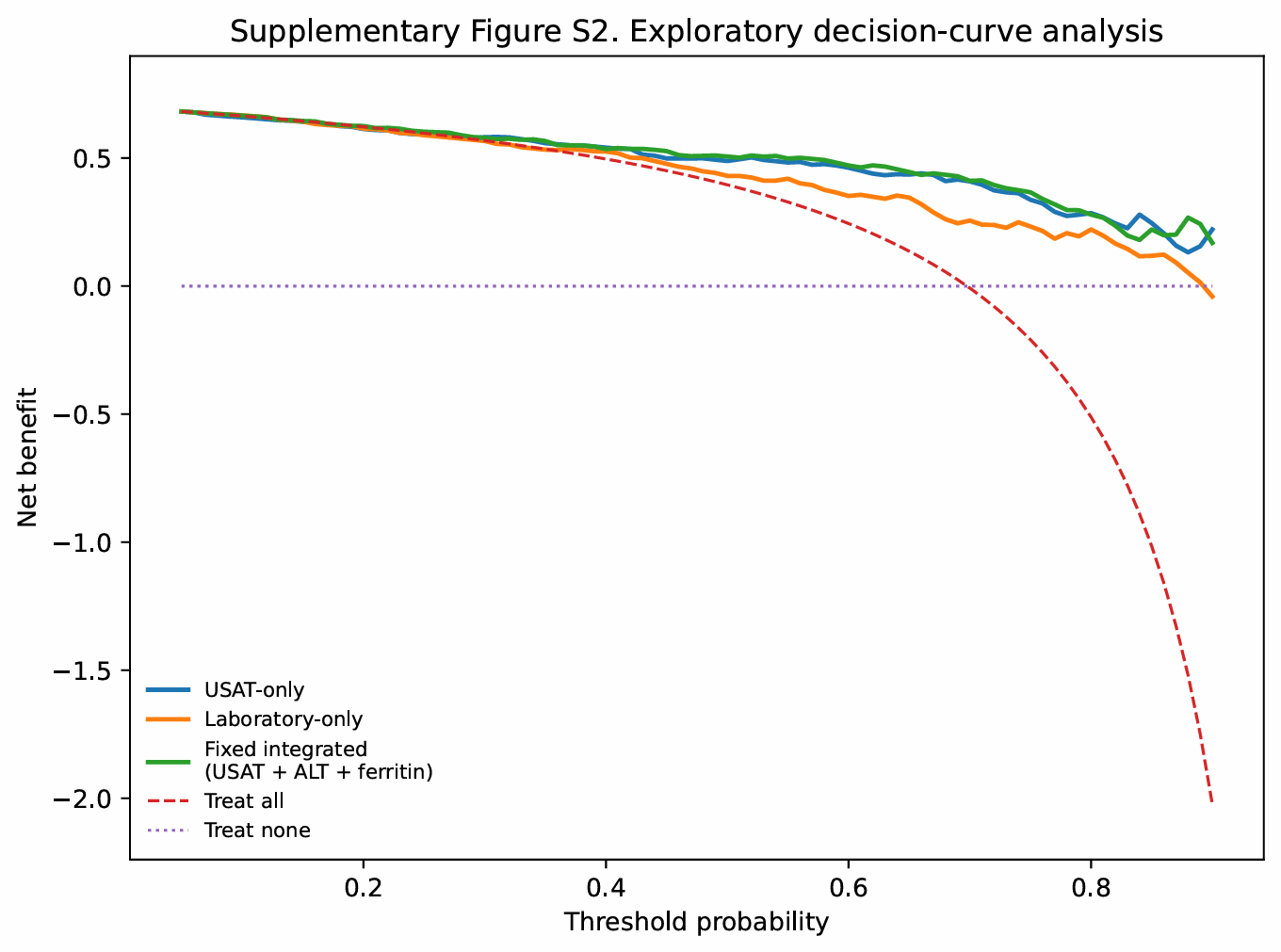


Net benefit curves were generated for the USAT-only, laboratory-only, and fixed integrated (USAT + ALT + ferritin) models across a range of threshold probabilities. The decision-curve analysis was interpreted as exploratory because the study was internally validated only and used QCT-derived comparator labels rather than an external clinical outcome.
