# Supplementary material for: Integrated model based on ultrasound attenuation and metabolic biomarkers for noninvasive assessment of hepatic fat fraction categories in MASLD: a QCT-referenced study
Source: Front Physiol. 2026 May 29;17:1804061. doi: 10.3389/fphys.2026.1804061 (PMC13259794; doi:10.3389/fphys.2026.1804061)
Supplement: Supplementary file 5 [file Table1.docx]

Supplementary Table S1. Model complexity and events-per-variable assessment

| **Analysis** | **Outcome** | **Predictors** | **Smallest relevant group** | **Approximate cases per predictor** | **Interpretation** |
| --- | --- | --- | --- | --- | --- |
| Laboratory-only benchmark | Binary QCT ≥1 vs QCT 0 | 17 | 52 QCT-negative cases | 3.1 | Exploratory benchmark; high risk of overfitting |
| USAT-only model | Binary QCT ≥1 vs QCT 0 | 1 | 52 QCT-negative cases | 52.0 | Parsimonious model |
| Fixed integrated model | Binary QCT ≥1 vs QCT 0 | 3 | 52 QCT-negative cases | 17.3 | Prespecified reduced model |
| Adjusted ordinal model | QCT categories 0–3 | 5 | 31 Category 2 cases | 6.2 | Interpreted cautiously |
| Three-class RF integrated model | Categories 1–3 | 3 | 31 Category 2 cases | 10.3 | Exploratory multiclass analysis |

Note-EPV = events per variable; QCT = quantitative computed tomography; USAT = ultrasound attenuation; RF = random forest. For binary models, the smaller outcome group was used to provide a conservative estimate of cases per predictor. For multiclass and ordinal models, the smallest category was used to reflect the most constrained class size.
